# Nanobodies against the myelin enzyme CNPase as tools for structural and functional studies

**DOI:** 10.1111/jnc.16274

**Published:** 2024-12-10

**Authors:** Sigurbjörn Markusson, Arne Raasakka, Marcel Schröder, Shama Sograte‐Idrissi, Amir Mohammad Rahimi, Ommolbanin Asadpour, Henrike Körner, Dmitri Lodygin, Maria A. Eichel‐Vogel, Risha Chowdhury, Aleksi Sutinen, Gopinath Muruganandam, Manasi Iyer, Madeline H. Cooper, Maya K. Weigel, Nicholas Ambiel, Hauke B. Werner, J. Bradley Zuchero, Felipe Opazo, Petri Kursula

**Affiliations:** ^1^ Department of Biomedicine University of Bergen Bergen Norway; ^2^ Neurosurgery Department Stanford University School of Medicine Stanford California USA; ^3^ Center for Biostructural Imaging of Neurodegeneration (BIN) University of Göttingen Medical Center Göttingen Germany; ^4^ Department for Neuroimmunology and Multiple Sclerosis Research University of Göttingen Medical Center Göttingen Germany; ^5^ Department of Neurogenetics Max Planck Institute for Multidisciplinary Sciences Göttingen Germany; ^6^ Faculty of Biochemistry and Molecular Medicine & Biocenter Oulu University of Oulu Oulu Finland; ^7^ VIB‐VUB Center for Structural Biology Vlaams Instituut voor Biotechnologie Brussels Belgium; ^8^ Department of Bioengineering Sciences, Structural Biology Brussels Vrije Universiteit Brussel Brussels Belgium; ^9^ Institute of Neuro‐ and Sensory Physiology University Medical Center Göttingen Göttingen Germany; ^10^ NanoTag Biotechnologies GmbH Göttingen Germany

**Keywords:** CNPase, function, imaging, myelin, nanobody, structure

## Abstract

2′,3′‐Cyclic nucleotide 3′‐phosphodiesterase (CNPase) is an abundant constituent of central nervous system non‐compact myelin, and its loss in mice and humans causes neurodegeneration. Additionally, CNPase is frequently used as a marker antigen for myelinating cells. The catalytic activity of CNPase, the 3′‐hydrolysis of 2′,3′‐cyclic nucleotides, is well characterised in vitro, but the in vivo function of CNPase remains unclear. CNPase interacts with the actin cytoskeleton to counteract the developmental closure of cytoplasmic channels that travel through compact myelin; its enzymatic activity may be involved in adenosine metabolism and RNA degradation. We developed a set of high‐affinity nanobodies recognising the phosphodiesterase domain of CNPase, and the crystal structures of each complex show that the five nanobodies have distinct epitopes. One of the nanobodies bound deep into the CNPase active site and acted as an inhibitor. Moreover, the nanobodies were characterised in imaging applications and as intrabodies, expressed in mammalian cells, such as primary oligodendrocytes. Fluorescently labelled nanobodies functioned in imaging of teased nerve fibres and whole brain tissue sections, as well as super‐resolution microscopy. These anti‐CNPase nanobodies provide new tools for structural and functional studies on myelin formation, dynamics, and disease, including high‐resolution imaging of nerve tissue.

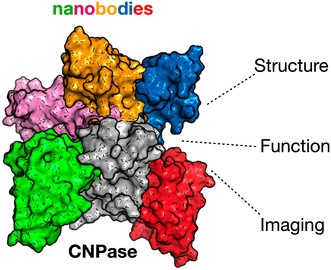

## Graphical abstract

The enzyme 2′,3′‐cyclic nucleotide 3′‐phosphodiesterase (CNPase) is abundant in non‐compact myelin. While its in vitro catalytic activity is well understood, the in vivo function of CNPase remains unclear at the molecular level. We developed high‐affinity nanobodies targeting the phosphodiesterase domain of CNPase, with crystal structures revealing distinct epitopes. Anti‐CNPase nanobodies are useful for understanding the structure and function of myelin molecules, as well as in imaging applications, including super‐resolution microscopy. The nanobodies provide new tools for research on myelin biology and related diseases.

**Abbreviations**
CDR: complementarity‐determining region
CNPase: 2′,3′‐cyclic nucleotide 3′‐phosphodiesterase
CNS: central nervous system
CV: column volume
DPBS: Dulbecco's phosphate‐buffered saline
DSF: differential scanning fluorimetry
EGFP: enhanced green fluorescent protein
hipp: hippocampus
iNb: intrabody
ITC: isothermal titration calorimetry
LUT: lookup table
MaBP: maltose‐binding protein
MBP: myelin basic protein
Nb: nanobody
NbCNP: anti‐CNPase nanobody
PBS: phosphate‐buffered saline
PDB: Protein Data Bank
PFA: paraformaldehyde
PNS: peripheral nervous system
ROI: region of interest
RRID: Research Resource Identifier
RT: room temperature
SAXS: small‐angle X‐ray scattering
STED: Stimulated Emission Depletion microscopy
TCEP: tris(2‐carboxyethyl)phosphine
VHH: single variable domains on a heavy chain

## 1 INTRODUCTION

Myelin is a specialised multilamellar membrane that ensheathes the axons of neurons in the central (CNS) and peripheral nervous system (PNS). It plays a crucial role in facilitating rapid and efficient transmission of electrical signals between neurons, enabling the functioning of the vertebrate nervous system (Nave & Werner, 2014). Myelin is formed by specialised glial cells, oligodendrocytes in the CNS and Schwann cells in the PNS. These cells wrap their plasma membrane around the axon, creating a multilayered proteolipid sheath (Mobius et al., 2008; Ruskamo et al., 2022). The myelin sheath is not continuous around a single axon, but rather segmented into internodes, separated by gaps called nodes of Ranvier. Disruptions in myelin formation or maintenance can lead to a variety of neurological disorders, which can range from mild to severe, affecting various aspects of nervous system function.

CNPase, or 2′,3′‐cyclic nucleotide 3′‐phosphodiesterase, is an enzyme of the 2H phosphodiesterase family (Myllykoski et al., 2016; Raasakka & Kursula, 2014; Sakamoto et al., 2005), highly expressed in myelinating cells (Gargareta et al., 2022; Lappe‐Siefke et al., 2003). Deficiency of CNPase in mice causes ultrastructural defects of the axon/myelin‐unit (Edgar et al., 2009; Lappe‐Siefke et al., 2003) and impairs the initiation of executive functions (Hagemeyer et al., 2012; Janova et al., 2018). A homozygous missense mutation in the human gene encoding CNPase correlates with white matter loss and associated neurodegeneration (Al‐Abdi et al., 2020), and *CNP* gene mutations in dogs have been linked to lysosomal storage disease and myelin abnormalities (Bullock et al., 2022; Keller et al., 2024). The catalytic function of the N‐terminal polynucleotide kinase‐like domain is controversial (Myllykoski, Itoh, et al., 2012; Raasakka & Kursula, 2014; Stingo et al., 2007), while the reaction catalysed by the C‐terminal phosphodiesterase domain has been known in vitro for >60 years (Drummond et al., 1962) and characterised mechanistically in detail (Myllykoski et al., 2013; Myllykoski, Raasakka, et al., 2012; Raasakka et al., 2015). It is unclear if the physiological function of CNPase is related to its enzymatic properties, or if CNPase has evolved to be more relevant through its molecular interactions with other proteins (Myllykoski, Itoh, et al., 2012; Snaidero et al., 2017), RNA (Gravel et al., 2009; Myllykoski, Raasakka, et al., 2012; Schwer et al., 2008), and lipid membranes (Bifulco et al., 2002; Esposito et al., 2008).

Nanobodies (Nbs), also known as single‐domain antibodies or single variable domains on a heavy chain (VHH), are small antigen‐binding fragments derived from heavy‐chain‐only antibodies found in camelids and cartilaginous fishes. Unlike conventional antibodies, which consist of two heavy and two light chains, Nbs are composed of a single VHH that has a strong, specific antigen‐binding capacity (Moliner‐Morro et al., 2020; Sockolosky et al., 2016). Nanobodies can be produced recombinantly in monomeric form (Hassanzadeh‐Ghassabeh et al., 2013; Muyldermans, 2013), further allowing for the easy generation of genetically modified variants and fusion constructs. Their small size and high stability make Nbs suitable for a wide range of applications, from structural to functional studies (Godoy Munoz et al., 2024; Gotzke et al., 2019; Ishizuka et al., 2022) to the development of diagnostics and treatments (Monti et al., 2023; Ruiz‐Lopez et al., 2022), as well as in advanced microscopy (Oleksiievets et al., 2022; Queiroz Zetune Villa Real et al., 2023).

We generated and characterised a set of five high‐affinity anti‐CNPase nanobodies (NbCNP) that can be used in various applications, including structural and functional studies. All NbCNPs were co‐crystallised with the catalytic domain of mouse CNPase, allowing the atomic‐level identification of five different antigenic epitopes on the CNPase surface. One Nb bound deep into the CNPase active site, acting as an inhibitor, while another one activated CNPase slightly. The high affinity of the NbCNPs allowed further validation in the imaging of nerve tissue using confocal and Stimulated Emission Depletion (STED) microscopy, and they could be expressed in cultured oligodendrocytes; in addition, their function as intrabodies in living cells was confirmed. The anti‐CNPase nanobodies provide state‐of‐the‐art tools for structural biology, as well as high‐resolution imaging of myelinated tissue and functional intervention of CNPase‐related processes.

## 2 MATERIALS AND METHODS

### 2.1 Statistics

No statistical methods were employed to predetermine sample size, and randomisation or blinding were not employed, unless otherwise mentioned below. Normality of the data for statistical purposes was not assessed, unless mentioned otherwise. Outliers in small‐angle X‐ray scattering (SAXS) data were removed using standard protocols in automated data processing pipelines; multiple X‐ray exposures of the sample were automatically compared, and only the frames without radiation damage were averaged and used further. Fits of molecular models to the raw SAXS data (*χ* ^2^ values) were calculated by the specified modelling programs. Crystallographic data collection and refinement statistics were calculated using standard protocols in the field, and no abnormalities in crystallographic reflection intensity distribution were detected. Specific statistical procedures and tests for individual experiments are described below in more detail.

### 2.2 Synaptosome preparation

Two adult male wild‐type Wistar RjHan:WI (RRID:RGD_13508588) rats (*Rattus norvegicus*) were obtained from the University Medical Center Göttingen and handled according to the specifications of the University of Göttingen and the local authority, the State of Lower Saxony (Landesamt für Verbraucherschutz, LAVES, Germany). The local authority (Lower Saxony State Office for Consumer Protection and Food Safety) approved the animal experiments (licence number: T09/08). The two male rats were kept in one Opti‐Rat IVC cage under a 12 h light/dark cycle with food and water available ad libitum. Rats were euthanised with CO_2_ followed by cervical dislocation, and brains were extracted to generate synaptosomes as described previously (Fornasiero et al., 2018). Briefly, brains were homogenised in precooled sucrose buffer (320 mM sucrose, 5 mM HEPES, pH 7.4). After centrifugation at 1000 *g* for 2 min, the supernatant was further centrifuged at 15 000 *g* for 12 min. A discontinuous Ficoll density gradient was applied. The fractions at the interface of 9% Ficoll were pooled, washed in sucrose buffer, and used for immunisation.

### 2.3 Immunisation

Two alpacas were immunised with enriched rat synaptosomes. The procedure was performed by Preclinics GmbH (Potsdam, Germany). Six injections were performed weekly with 0.5 mg of protein from enriched synaptosomes. Two weeks after the last immunisation, a single boost with 0.5 mg of synaptosomes was performed, and 100 mL of blood was taken 3 and 5 days after the boost immunisation. Peripheral blood mononuclear cells (PBMCs) were isolated using a Ficoll gradient, and the remaining serum was stored at −80°C. Total RNA was extracted using an RNA extraction kit (Qiagen; RRID:SCR_008539).

### 2.4 Enrichment of IgG2 & IgG3 from plasma

Plasma from the two fully immunised animals was enriched in IgG2 and IgG3 following the original protocol (Queiroz Zetune Villa Real et al., 2023). Filtered plasma was injected into a HiTrap protein G HP (Cytiva; RRID:SCR_023581) column, and the flowthrough was collected and injected into a HiTrap protein A (Cytiva) column. Bound IgGs on the protein G column were eluted first at pH 3.5, followed by a second elution using pH 2.7. The IgG bound to the protein A column was eluted at pH 4.0. Eluted fractions were neutralised using 1 M Tris–HCl, pH 9.0. The fractions were analysed on denaturing SDS‐PAGE, and IgG2 and IgG3 fractions were pooled.

### 2.5 Plasma‐ELISA

The purified full‐length human and mouse CNPase (hCNPase and mCNPase) were immobilised overnight at +4°C on a 96‐well immunosorbent plate (Nunc). All the following steps were done by gentle shaking on an orbital shaker. Wells were washed with phosphate‐buffered saline (PBS) and blocked with 5% (w/v) skim milk in PBS for 3 h at room temperature (RT). After rinsing the wells with PBS, the enriched IgG2‐IgG3 mixture was added at a concentration of 0.5 μg/μL and incubated on the wells at RT. Bound IgG2 and IgG3 were then revealed with a monoclonal mouse anti‐camelid antibody coupled to HRP (Preclinics, clone: P17Ig12) diluted 1:2000 in PBS. The ELISA was revealed by adding 100 μL of TMB substrate (ThermoScientific) until the blue colour was stable. The reaction was quenched with 100 μL of 2 M sulphuric acid. The absorbance was read at 430 nm (BioTek Cytation).

### 2.6 Nanobody library generation

Total mRNA was extracted from the enriched PBMC from the 2 alpacas using a standard RNA extraction kit (Qiagen; RRID:SCR_008539). As described (Queiroz Zetune Villa Real et al., 2023), the recovered mRNA was retrotranscribed to cDNA using Superscript IV (Invitrogen) and the Cal 0001/2 primers. PCR products were diluted to 5 ng/μL, and 1.5% agarose gel electrophoresis was used to confirm the size of the PCR product. The obtained phagemids were purified using a PCR purification kit (Qiagen). The library was then electroporated into TG1 bacteria. For the transformation, 65 ng of DNA was added to 50 μL of TG1 and repeated 20 times. The electroporated bacteria were left 1 h at +37°C, then pooled together in 400 mL of 2YT medium (ThermoFisher; RRID:SCR_008452) supplemented with antibiotics and cultured overnight at +37°C. The next day, bacteria were pelleted and resuspended in 25 mL LB medium (ThermoFisher; RRID:SCR_008452) containing 25% glycerol. The library was aliquoted, snap‐frozen in liquid nitrogen, and stored at −80°C.

### 2.7 Phage display

A 1‐mL library aliquot was grown in 500 mL of 2YT medium supplemented with antibiotic and grown at +37°C until OD_600_ reached ca. 0.5. Next, ca. 1 × 10^12^ M13KO7 Helper Phages (NEB) were added to the culture, allowing infection for 45 min. Infected bacteria were incubated overnight at +30°C to produce phages. The next day, the culture supernatant was incubated with 4% (w/v) PEG8000 and left on ice for <2 h to allow phages to precipitate. After several washes in PBS, phages were filtered through a 0.45‐μm syringe filter (Sartorius; RRID:SCR_003935). Full‐length human and mouse CNPase was conjugated to desthiobiotin‐*N*‐hydroxysuccinimide ester (Berry and Associates). 1–3 nmol of mixed antigen was bound to pre‐equilibrated Dynabeads MyOne Streptavidin C1 (ThermoFisher; RRID:SCR_008452). The purified phages were mixed with the beads pre‐loaded with a combination of hCNPase and mCNPase and incubated for 2 h at RT. Beads were thoroughly washed in PBS‐T. CNPase and bound phages were eluted using 50 mM biotin in PBS. The eluted phages were used to reinfect TG1 cells and initiate another panning cycle. After the last panning round, bacteria were plated on LB agar supplemented with antibiotics, and the following day, 96 colonies were picked and grown in 96‐deep‐well plates.

### 2.8 Phage and ramp ELISAs

Each clone in the 96‐deep‐well plate was infected with helper phages and incubated overnight. Bacteria were centrifuged down, and supernatants containing phages were used directly. The desthio‐biotinylated mCNPase and hCNPase were immobilised for 2 h at RT on streptavidin‐coated flat‐bottom 96‐well plates (Thermo Fisher Scientific). The wells were washed three times 10 min with PBS and blocked using 5% skim milk in PBS‐T for 3 h at RT. Next, 25 μL of phages from each well were incubated with immobilised hCNPase or mCNPase 2 h at RT. Bound phages were detected by staining for 1 h with anti‐major coat protein M13‐HRP (Santa Cruz; RRID:AB_673750) diluted 1:1000 in 100 μL PBS. TMB substrate was added to each well, and the colorimetric reaction was stopped with 100 μL of 2 M sulphuric acid. The absorbance was read at 430 nm using a plate reader (BioTek Cytation; RRID:SCR_019731).

### 2.9 Tissue imaging with anti‐CNPase nanobodies

#### 2.9.1 Nanobody expression and purification for imaging applications

Nanobodies for imaging were produced in SHuffle® Express (NEB; RRID:SCR_013517) cells, using a vector coding for a His_14_ tag and bdSUMO as fusion partners (Frey & Gorlich, 2014; Maidorn et al., 2019). Bacteria were grown in Terrific Broth supplemented with kanamycin at +30°C. When OD_600_ reached ca. 3, 0.4 mM IPTG was added. Induction was allowed for ca. 16 h. Cultures were centrifuged, and the pellet was resuspended in cold lysis buffer (LysB: 100 mM HEPES, 500 mM NaCl, 25 mM imidazole, 2.5 mM MgCl_2_, 10% v/v glycerol, 1 mM DTT, pH 8.0) supplemented with 1 mM PMSF. After disruption by sonication, the lysate was centrifuged at ca. 11 000 g for 1.5 h at +4°C. The supernatant was incubated with LysB‐equilibrated Ni^2+^ beads (cOmplete, Roche) for 1 h at +4°C. Beads were washed with 3 column volumes (CV) using LysB buffer and with 5 CV of high salt buffer (HSB; 50 mM HEPES, 1.5 M NaCl, 25 mM imidazole, 2.5 mM MgCl_2_, 5% v/v glycerol, 1 mM DTT, pH 7.5). Finally, beads were washed in the buffer of choice for the next application. Elution was carried out using bdSENP1 protease cleavage on the column (Frey & Gorlich, 2014). Eluted nanobodies were evaluated on SDS‐PAGE.

#### 2.9.2 Fluorophore conjugation

Purified nanobodies bearing an ectopic cysteine (at their C‐terminus or N‐ and C‐termini) were reduced for 1 h on ice using 10 mM tris(2‐carboxyethyl)phosphine (TCEP). Excess TCEP was removed using a NAP‐5 column (GE Healthcare; RRID:SCR_000004) pre‐equilibrated with cold‐degassed PBS, pH 7.4. Freshly reduced nanobodies were immediately mixed with ca. 3‐5‐fold molar excess of maleimide‐functionalised fluorophore and incubated for 2 h at RT. Excess dye was removed using a Superdex™ 75 increase 10/300 GL column (Cytiva) on the Äkta‐Prime FPLC system (RRID:SCR_019958).

#### 2.9.3 Histology and immunohistochemistry

Two male 20‐week‐old C57BL/6 mice (RRID:MGI:2159769) were sacrificed using CO_2_ and intracardially perfused with saline (5 min) followed by a fixative containing 4% paraformaldehyde (PFA; 10 min). Up to five mice were housed in IVC cages under a 12 h light/dark cycle with food and water available ad libitum. Mouse brains were post‐fixed in 4% PFA for 24 h, transferred into 30% sucrose, and embedded in OCT. CNPase staining was performed on frozen sections. Tissue sections were first blocked and permeabilised with 10% (v/v) FCS and 0.2% (v/v) Tween‐20 for 30 min at RT. The following fluorescently labelled nanobodies were used for overnight incubation at +4°C: Nb5E‐Star635p, Nb8D‐Star635p, and Nb10E‐Star635p at a concentration of 20 nM. Sections were washed in PBS/0.05% (v/v) Tween‐20, stained with DAPI (1 μg/mL, Sigma‐Aldrich, cat. no. MBD0015), and embedded with Fluoromount‐G (SouthernBiotech, cat. no. 0100‐01).

#### 2.9.4 Nerve preparation

The *Cnp*‐wildtype and *Cnp*‐null alleles were discriminated by genomic PCR as described (Lappe‐Siefke et al., 2003), using DNA extracted from ear punch biopsies. For immunolabelling of teased fibre preparations, sciatic nerves dissected from two mice were transferred into ice‐cold PBS and processed as described (Eichel et al., 2020). Briefly, mice in the C57Bl/6 background were bred and kept in IVC cages with 12 h dark/light cycle with food and water available ad libitum in the mouse facility of the Max Planck Institute of Multidisciplinary Sciences registered according to §11 Abs. 1 TierSchG and sacrificed by cervical dislocation in accordance with the German animal protection law (TierSchG) and approved by the Niedersächsisches Landesamt für Verbraucherschutz und Lebensmittelsicherheit (LAVES) under licence 33.19‐42502‐04‐17/2409. Using two fine forceps (Dumont No. 5), the epineurium was removed from the dissected sciatic nerves, and small nerve pieces were transferred onto a new coverslip. By pulling the fibre bundles carefully apart with both forceps, the axons were separated from each other. Slides were dried and stored at −20°C for later immunolabelling.

#### 2.9.5 Antibodies and staining reagents

Recombinant rat anti‐myelin basic protein (MBP; Abcam cat. no. ab7349, 1:20000 dilution; RRID:AB_305869) and mouse anti‐CNPase (Millipore cat. no. MAB326R, 1:100 dilution; RRID:AB_94780) were detected by the following secondary antibodies (used at a 1:1000): donkey anti‐rat AlexaFluor 488 (Invitrogen cat. no. A‐21208; RRID:AB_141709) and donkey anti‐mouse Alexa Fluor 488 (Invitrogen cat. no. A‐21202; RRID:AB_141607) were used for Figure 9 on cultured oligodendrocytes. Filamentous actin was stained using phalloidin AlexaFluor 488 conjugate (Invitrogen cat. no. A12379) at a 7:1000 dilution. HCS CellMask Blue (Thermo Scientific cat. no. H32720) staining was used at 1:1000 dilution to distinguish intact cells from cell debris. Rabbit polyclonal anti‐NaV1.6 (Alomone labs, cat. no. ASC‐009; RRID:AB_2040202) was used in 1:100 dilution followed by 1:500 dilution of a secondary nanobody fused to AberriorStar580 (NanoTag Biotechnologies, cat. no. N2402) in the STED experiment, as shown in Figure 6. Fluorescently labelled anti‐CNPase nanobodies described in this manuscript were used at ca. 20 nM.

#### 2.9.6 Immunofluorescence

Teased fibres were blocked and permeabilised with 3% (w/v) BSA, 0.1% (v/v) Triton X‐100 for 20 min at RT with gentle shaking. Nanobodies (see details below on Antibodies and staining reagents) were applied in PBS supplemented with 1.5% BSA and 0.05% Triton X‐100 for 1 h at RT with gentle shaking. When indirect detection was needed, secondary nanobodies were added for 1 h at RT, followed by several PBS washes and DAPI staining (1:1000 dilution Sigma‐Aldrich, cat. no. MBD0015). Coverslips were rinsed in distilled water and mounted using Mowiöl (12 mL of 0.2 M Tris buffer pH 7.5, 6 mL distilled water, 6 g glycerol, 2.4 g Mowiöl 4–88, Merck Millipore). Samples were imaged immediately or within the next 48 h, and samples were kept at +4°C.

Staining of cultured oligodendrocytes for Figure 9 was performed on day 5 of differentiation; the cell medium was removed by suction, and cells were fixed in 4% PFA in PBS for 15 min, followed by three PBS washes. Cells were permeabilised using 0.1% Triton X‐100 in PBS for 3 min, followed by three PBS washes. Before primary staining, cells were incubated for 30–60 min in 3% BSA in PBS. All procedures were performed at RT. Primary antibodies were diluted in 3% BSA in PBS and were incubated overnight at +4°C. The next day, three PBS washes and the addition of diluted AlexaFluor 488‐conjugated secondary antibodies (anti‐rat for MBP and anti‐mouse for CNPase staining) were carried out. Secondary staining was performed for 1 h at RT, followed by three PBS washes. All cells were finally stained using diluted HCS CellMask Blue, followed by three PBS washes. For actin staining, primary and secondary antibody staining were omitted, and a diluted solution of phalloidin AlexaFluor 488 conjugate in PBS containing diluted HCS CellMask Blue was applied for 15 min, followed by three PBS washes. Coverslips were mounted with Fluoromount G (SouthernBioTech, cat. no. 0100‐20). Hardening was allowed overnight at room temperature.

#### 2.9.7 Epifluorescence microscopy

For Figure 6, images were taken by an inverted Nikon Ti epifluorescence microscope (Nikon Corporation, Japan; RRID:SCR_021242) equipped with a Plan Apochromat 60×,1.4 NA oil immersion objective, an IXON X3897 Andor camera controlled by NIS‐Elements software. Excitation was achieved using a Mercury lamp with an ND filter of 8, and exposure was 50 ms for DAPI and 200 ms for Cy5 channel (for NbX‐Ab635p). For Figure 7, images of brain slices were acquired with a VS120‐L100‐J slide scanner (Olympus, VS‐ASW software) equipped with an X‐Cite exacte DC‐powered mercury lamp using a 10x objective. Excitation power and exposure times for DAPI and AberriorStar635p (Nbs) were automatically selected by the instrument at the beginning and kept the same through the images of the following sample for direct comparison. For Figure 9, A Zeiss Axio Observer Z1 microscope (RRID:SCR_021351) was used for epifluorescence imaging using a Plan‐Neofluar 40×/1.30 Oil DIC objective through Zeiss Immersol 518 F immersion oil (*n*_e_ = 1.518 (23°C)) at ambient temperature. Exposure times were 20 ms for HCS CellMask Blue, 100 ms for AlexaFluor 488, and 1 s for mRuby3. Images were acquired blinded for construct using identical illumination and acquisition settings.

#### 2.9.8 Confocal and STED microscopy

Images from mounted samples in Mowiöl were acquired using a STED Expert Line microscope (Abberior Instruments, Göttingen, Germany) for Figures 6c and 8. The microscopy setup comprised an IX83 inverted microscope (Olympus, Hamburg, Germany; RRID:SCR_020344) equipped with UPLSAPO 100× 1.4 NA oil immersion objective (Olympus). In addition, 488, 561, and 640 nm lasers were used for confocal imaging. High‐resolution images were obtained using the 775 nm pulsed STED depletion laser from the same setup. Pixel size was 60 and 20 nm for confocal and STED, respectively. Excitation on the sample was achieved using 1 μW of energy at the sample. STED depletion beam was used at 1 mW at the sample. Dwelling times per pixel were 10 μs. Confocal has no line accumulation; for STED, 3× lines were accumulated. Images were analysed in Fiji/ImageJ (v. 1.53o; RRID:SCR_002285).

### 2.10 Expression of anti‐CNPase nanobodies as intrabodies

#### 2.10.1 Construct preparation

Mammalian expression plasmids encoding fluorescent NbCNP constructs were created using InFusion cloning (Takara Bio; RRID:SCR_021372). DNA fragments encoding the open reading frame of each NbCNP with 15‐bp complementary overhangs were amplified using PCR and purified from agarose gel bands (Macherey‐Nagel cat. no. 740609.50). A pAAV vector, containing a CMV promoter, the open‐reading frame of mRuby3 (Bajar et al., 2016), and a hGH polyA sequence, was linearised using *Age*I‐HF and *Xcm*I (New England Biolabs) digestion and purified from an agarose gel band. Linear NbCNP DNA fragments were mixed with the linearised vector and cloned in InFusion reactions, followed by transformation to OneShot Stabl3 chemically competent cells (Fisher Scientific cat. no. C737303), plasmid propagation and purification, and DNA sequencing (Sequetech Corporation, Mountain View, CA). The obtained constructs had the architecture CMV‐NbCNP‐mRuby3‐hGH polyA, where the nanobody and mRuby3 are linked by the amino acid sequence GDPPVAT. The constructs were amplified in OneShot Stabl3 cells and purified using a plasmid midi kit (Qiagen cat. no.12945) for high yields of endotoxin‐free DNA. TOM70‐EGFP‐CNPase‐ALFAtag was ordered as a synthetic gene (GeneArt Thermo Scientific) and cloned into the mammalian expression plasmid pcDNA3.1 using Gibson assembly (NEB). Constructs were validated by Sanger sequencing.

#### 2.10.2 Culture and transformation COS‐7 cells

COS‐7 fibroblasts (RRID:CVCL_0224) were obtained from the Leibniz Institute DSMZ—German Collection of Microorganisms and Cell Culture (DSMZ Braunschweig, Germany; RRID:SCR_001711) and cultured in Dulbecco's MEM supplemented with 10% FBS, 4 mM L‐glutamine, 0.6% penicillin and streptomycin, at +37°C, 5% CO_2_ in a humidified incubator. For immunostaining, cells were plated on poly‐L‐lysine‐coated coverslips in 12‐well plates. Cells on coverslips were transfected and co‐transfected using 500 ng of each plasmid mixed with 2 μL of Lipofectamine 2000 (ThermoFisher) per coverslip. Cells were fixed using 4% PFA + 0.025% glutaraldehyde ca. 16 h after being transfected. Fixed cells were stained with DAPI (1:1000 dilution Sigma‐Aldrich, cat. no. MBD0015) and mounted on Mowiöl (12 mL of 0.2 M Tris buffer, 6 mL distilled water, 6 g glycerol, 2.4 g Mowiol 4–88, Merck Millipore cat. no. 475904; RRID:SCR_008983) for imaging.

#### 2.10.3 Oligodendrocyte progenitor cell purification

Oligodendrocyte progenitor cells (OPCs) were purified using an immunopanning protocol from P5‐P7 Sprague Dawley rat (RRID:MGI:5651135) brains as described (Dugas & Emery, 2013). The P5‐P7 rat pups were sacrificed by decapitation under no anaesthesia. The involved animal procedures were approved by the Institutional Administrative Panel on Laboratory Animal Care (APLAC) of Stanford University (RRID:SCR_023388) and followed the National Institutes of Health guidelines under animal protocol APLAC 32260. The freshly purified primary OPCs were plated onto ∅10‐cm tissue culture plates (Fisher Scientific cat. no. 08‐772E; coated with 10 μg/mL poly‐d‐lysine, Sigma‐Aldrich cat. no. P6407) in OPC proliferation media (DMEM‐Sato) supplemented with 10 ng/mL recombinant human ciliary neurotropic factor (CNTF; Peprotech cat. no. 450‐13; RRID:SCR_006802), 10 μM forskolin (Sigma‐Aldrich cat. no. F6886), 10 ng/mL recombinant human platelet‐derived growth factor AA (PDGF; Peprotech cat. no. 100‐13A) and 1 ng/mL recombinant human neutrophin 3 (NT‐3; Peprotech cat. no. 450‐03) and allowed to recover and proliferate at +37°C/10% CO_2_. Half of the media was replenished every 2 days, and cell morphology and density were monitored daily until transfection.

#### 2.10.4 Transfection and differentiation of oligodendrocyte progenitor cells

Proliferating OPCs were detached from plates using 0.0025% trypsin (Gibco cat. no. 25300120; diluted using Earle's balanced salt solution, Sigma‐Aldrich cat. no. E6267) and 30% fetal bovine serum (Gibco cat. no. 10437028; diluted in Dulbecco's phosphate‐buffered saline (DPBS), Cytiva HyClone cat. no. SH30264.01), and collected via centrifugation at 90 g for 10 min. For transfection, OPCs were gently resuspended in P3 Primary Cell Nucleofector solution (Lonza cat. no. V4XP‐3032) at 10 000–12500 cells/μL. For each transfection reaction, 20 μL of cell suspension was mixed with 400 ng of endotoxin‐free plasmid DNA. Electroporation was performed using a Lonza 4D‐Nucleofector X Unit (cat. no. AAF‐1003X; RRID:SCR_023155) assembled with a 4D‐Nucleofector Core Unit (cat. no. AAF‐1002B) with pulse code DC‐218. The electroporated cells were allowed to rest for 10 min at ambient temperature, after which 80 μL of antibiotic‐free DMEM‐Sato base media was added to each electroporated cell suspension. Each diluted suspension was carefully mixed and further diluted to 2 mL using OPC differentiation media (antibiotic‐free DMEM‐Sato supplemented with 10 ng/mL CNTF (Peprotech cat. no. 450‐13), 10 μM forskolin (Sigma‐Aldrich cat. no. F6886) and 40 ng/mL 3,3′,5‐triiodo‐L‐thyronine (Sigma‐Aldrich cat. no. T6397)). From each suspension, 100 μL aliquots were transferred onto ∅12‐mm glass coverslips (coated with 10 μg/mL PDL in 1.5 mM boric acid, pH 8.4) in a 24‐well tissue culture plate (Falcon cat. no. 353047). The cells were allowed to adhere to the surface for 30 min at ambient temperature, after which 400 μL of OPC differentiation media was added to each well. The cells were differentiated for 5 days at +37°C/10% CO_2_, with half of the media replenished in each well on day 3 post‐transfection.

#### 2.10.5 Image analysis

Regions‐of‐interest (ROI) were defined in Fiji (Schindelin et al., 2012) around cells using pixel intensity thresholding and manual drawing, and fluorescence intensity values were extracted from images. Binary images of ROI masks were generated in Fiji and used to calculate Pearson's correlation coefficients (*r*‐values) in MatLab R2021b (RRID:SCR_001622). Statistical analyses were performed using GraphPad Prism (RRID:SCR_002798). Briefly, Pearson's *r*‐values from 6 to 44 cells were averaged for each biological replicate (*n* = 3 rat brains) and one‐way ANOVA with Dunnett's multiple comparisons test was used to calculate statistical significance in Figure 9b. Statistical significance of fluorescence intensity differences between mRuby3 control and NbCNP 10E was calculated using two‐tailed Student's *t*‐test in Figure S5f. Pearson's correlation coefficient distributions for the OPC experiment were tested for normality using the D'Agostino & Pearson, the Shapiro–Wilk, and the Kolmogorov‐Smirnov normality tests within GraphPad Prism. mRuby3 and 7E data were normally distributed; 8D and 10E were not, since the coefficients are quite high, but the values cannot exceed 1. This skewing towards the upper limit value of 1 had no effect on the conclusions (see also Table S3).

### 2.11 Recombinant CNPase production

Recombinant mCNPase variants (full‐length and catalytic domain) and full‐length hCNPase were expressed using pTH27 vectors (Hammarström et al., 2002) using autoinduction (Studier, 2005) in *E. coli* Rosetta (DE3) and purified as described (Myllykoski & Kursula, 2010). The purity of the main size exclusion chromatography (SEC) fractions was analysed via SDS‐PAGE; pure fractions were pooled and concentrated to 18–29 mg/mL. Full‐length CNPase was split into 50 μL aliquots, snap‐frozen in liquid N_2_, and stored at −80°C. The purified mCNPase catalytic domain was stored on ice.

### 2.12 Anti‐CNPase Nb production for structural studies

For large‐scale expression and purification for structural studies, NbCNP cDNAs were cloned into pTH27 (N‐terminal His_6_ tag; (Hammarström et al., 2002)) and the pHMGWA vector (N‐terminal His_6_‐maltose binding protein (MaBP); (Busso et al., 2005)), using the Gateway system (Invitrogen).

NbCNP 10E was expressed from pTH27 and purified like described for anti‐Arc Nbs (Markusson et al., 2022). TEV proteolysis was carried out for the His‐tagged protein after the SEC step, at +37°C for 2–4 h, and a reverse NiNTA (Qiagen; RRID:SCR_008539) affinity purification step was done to remove TEV protease and the cleaved tag. Final purification was carried out on a Superdex 75 10/300 Increase GL SEC column in 20 mM Tris–HCl pH 7.4, 150 mM NaCl. Fractions assessed pure via SDS‐PAGE were pooled, concentrated in a 10‐kDa MWCO spin concentrator, split into 50 μL fractions, snap‐frozen in liquid N_2_, and stored at −80°C.

Due to low yields, the other NbCNPs were cloned into the pHMGWA vector, resulting in N‐terminal His_6_‐MaBP fusion constructs. Competent *E. coli* Shuffle T7 cells (New England Biolabs, MA, USA; RRID:SCR_013517) were transformed with the pHMGWA‐MaBP‐Nb constructs. A single transformed colony was used to inoculate 10 mL of LB starter culture containing 100 μg/mL ampicillin and incubated overnight at +30°C and 200 rpm. The starter cultures were diluted 100‐fold into 500 mL of the same medium. The cultures were incubated at +30°C and 200 rpm until OD_600_ reached 0.5–0.7. Protein expression was induced with 0.4 mM IPTG at +20°C for 20 h. Cells were harvested via centrifugation at 6000 g and + 4°C for 1 h, supernatant discarded and pellets resuspended in 50 mM HEPES, 500 mM NaCl, 5 mM MgCl_2_, 25 mM imidazole, 10% (v/v) glycerol and 1 mM DTT, pH 8.0 (35 mL per 500 mL expression culture) supplemented with 0.1 mg/mL lysozyme and complete EDTA‐free protease inhibitors. Cells were lysed via a single freeze–thaw cycle followed by sonication, and the soluble fraction was collected via centrifugation at 30 000 g and +4°C for 1 h. The soluble fraction was filtered through 0.45 μm syringe filters and applied to a NiNTA agarose resin equilibrated in 50 mM HEPES, 500 mM NaCl, 5 mM MgCl_2_, 10% (v/v) glycerol and 1 mM DTT, pH 8.0 and the resin washed with 12 CV of the same buffer. Bound protein was eluted in 5 CV (10 mL) of the same buffer containing 500 mM imidazole. TEV protease was added to the eluate, followed by dialysis against 1 L of 50 mM HEPES, 500 mM NaCl, 5 mM MgCl_2_, 10% (v/v) glycerol, and 1 mM DTT (pH 8.0). As these constructs cleaved poorly, at least 3 mg of TEV protease were added to each 10 mL eluate, and dialysis was maintained at +4°C for 35 h, renewing the DTT in the buffer after the first 24 h. The proteins were then subjected to reverse NiNTA affinity purification to remove TEV protease, uncleaved fusion protein, and free affinity tag. The flowthrough and wash fractions were concentrated to 1 mL in 10 kDa MWCO spin concentrators and further purified on a Superdex 75 Increase 10/300 GL SEC column in 20 mM HEPES, 150 mM NaCl, 0.5 mM TCEP, pH 7.5. Fractions determined to contain pure Nb by SDS‐PAGE were pooled, concentrated to 5–25 mg/mL, and split into 50‐μL aliquots before snap‐freezing in liquid N_2_ and storing at −80°C. The identity of all purified nanobodies was confirmed by mass spectrometry. For structural work on NbCNP 8D, a protein batch from the His_14_‐bdSUMO fusion system (see above) was used, due to high yields of pure protein.

### 2.13 Folding and stability assays

Differential scanning fluorimetry (DSF), or Thermofluor (Ericsson et al., 2006), was used to assess the thermal stability of CNPase constructs upon Nb binding. The assay buffer was 20 mM HEPES, 150 mM NaCl, 0.5 mM TCEP and pH 7.5. Proteins were diluted to 0.5–2 mg/mL in the assay buffer and mixed with 100× SYPRO‐Orange (in 50% (v/v) DMSO/assay buffer) in 384‐well PCR plates to a final concentration of 5x SYPRO‐Orange, making the final DMSO concentration in the assay 2.5% (v/v). The assay volume was 18 μL. Fluorescence emission at 610 nm, following excitation at 465 nm, was measured in a LightCycler 480 LC RT‐PCR system (Roche, Basel, Switzerland; RRID:SCR_018626) over the temperature range of 20–95°C, with a temperature ramp of +2.4°C/min. *T*_m_ was determined as the maximum of the first derivative of the melting curve. If more than one prominent peak appeared in the first derivative plot, the identity of the main and secondary peaks was determined by the approximate area under the peak.

For nanobody 8D, the stabilisation of CNPase by the Nb was additionally studied with nanoDSF using a Prometheus NT.48 instrument (NanoTemper Technologies, Munich, Germany). NanoDSF is a label‐free method based on Trp fluorescence, and nanoDSF samples were at 2 mg/mL in SEC buffer (20 mM HEPES, 200 mM NaCl, 0.5 mM TCEP, pH 7.5). The samples included the nanobody, CNPase, and the complex prepared by SEC.

### 2.14 Pull‐down binding assay

For assessment of Nb binding and crude epitope mapping, 0.5 mg/mL of mCNPase catalytic domain were mixed with an equimolar amount of Nb and incubated on ice for 20–45 min. 200 μL of the sample were mixed with 100 μL of NiNTA agarose resin pre‐equilibrated in 20 mM HEPES, 150 mM NaCl, 20 mM imidazole, 0.5 mM TCEP, pH 7.5. The mixture was incubated at +4°C under gentle agitation for 1 h, followed by centrifugation at 200 g, +4°C for 5 min. The supernatant (unbound protein) was decanted, and the resin washed three times in the same buffer. To elute bound protein, the resin was incubated in the same buffer with 300 mM imidazole for 30 min before centrifugation as above. The fractions were analysed via SDS‐PAGE on precast 4–20% TGX gradient gels (Bio‐Rad Laboratories, Hercules, CA, USA, RRID:SCR_008426). His_6_‐tagged NbCNP‐10E was subjected to pull downs with untagged CNPase (full‐length and catalytic domain with and without C‐terminal tail) in the same manner.

### 2.15 Calorimetry

The thermodynamics and affinity of Nb binding to CNPase were measured on a MicroCal iTC200 instrument (Malvern Panalytical, Malvern, UK; RRID:SCR_018590). The binding of NbCNPs to mCNPase catalytic domain was measured in 20 mM HEPES, 150 mM NaCl, 0.5 mM TCEP, pH 7.5 with 8.5–11 μM CNPase in the cell and 88–95 μM Nb in the syringe at +25°C, with a reference power of 5 μcal/s and the same injection volumes and timing as above. Data analysis was carried out in Origin (RRID:SCR_002815). Binding enthalpy (∆H), association/dissociation constant (*K*_a_/*K*_d_), and binding entropy (∆S) were obtained through fitting to a 1:1 binding model.

### 2.16 Crystallisation and structure determination

The CNPase catalytic domain was co‐crystallised with each of the five NbCNPs using sitting‐drop vapour diffusion. Table 1 contains the specific methods for each complex. Diffraction data for the CNPase‐Nb complexes were collected using synchrotron radiation on the beamline P11 at PETRAIII/DESY (Hamburg, Germany; Burkhardt et al., 2016; Meents et al., 2013) at an X‐ray wavelength of 1.033 Å on a Dectris Eiger 16 M detector and processed using XDS (RRID:SCR_015652; Kabsch, 2010; Table 2). As the data for the NbCNP‐5E complex showed moderate anisotropy, anisotropic scaling was carried out for this dataset using STARANISO (http://staraniso.globalphasing.org/cgi‐bin/staraniso.cgi; Tickle et al., 2016; RRID:SCR_018362). Data quality was analysed in XTRIAGE (Liebschner et al., 2019) and molecular replacement done using PHASER (RRID:SCR_014219; McCoy et al., 2007). The search model for CNPase was Protein Data Bank (PDB; RRID:SCR_012820) entry 2XMI (Myllykoski, Raasakka, et al., 2012) and for each nanobody, the search model was a close sequence homologue (Table 1). Refinement was carried out in PHENIX.REFINE (RRID:SCR_014224; Afonine et al., 2012) and manual building in Coot (RRID:SCR_014222; Emsley et al., 2010). Structure validation was performed using MolProbity (RRID:SCR_014226; Chen et al., 2010). Table 1 lists specific details of structure determination for each complex, and Table 2 has the data processing and refinement statistics.

**TABLE 1:** Crystallisation and structure solution of CNPase‐nanobody complexes.

NbCNP	5E	7E	8C	8D	10E
Temperature (°C)	20	8	8	8	20
Well solution	0.2 M Na/K phosphate pH 7.0, 25% PEG3350	0.1 M MIB pH 4.0, 25% PEG3350	0.1 M MIB pH 8.0, 25% PEG1500	0.1 M phosphate–citrate pH 4.2, 18% PEG8000, 0.35 M NaCl	0.2 M Na malonate, 20% PEG3350
Protein concentration (mg/mL)	8.9	7.5	7.4	8.5	11.2
Cryoprotectant	25% PEG400	‐	25% PEG400	30% glycerol	25% PEG400
Nanobody model PDB entry	6GWN chain C (Sillen et al., 2020)	6H7J chain C (Warne et al., 2019)	6QV2 chain E (Hutter et al., 2019)	AlphaFold2 (Jumper et al., 2021) model of NbCNP‐8D	7KN7 chain B (Koenig et al., 2021)
Copies of complex in asymmetric unit	1	4	1	2	4

*Note*: MIB; buffer mixture of malonic acid, imidazole, and boric acid. Different cryoprotectants were tested for the crystals, and the condition used for data collection is shown for each complex.

**TABLE 2:** X‐ray diffraction data processing and crystal structure refinement.

NbCNP	5E	7E	8C	8D	10E
Data processing					
Space group	P3_1_2	P1	P2_1_	P1	P2_1_2_1_2_1_
Unit cell parameters	110.8, 110.8, 83.3 Å; 90, 90, 120°	37.2, 79.0, 117.6 Å; 77.7, 87.7, 84.3°	39.86, 54.17, 80.4 Å; 90, 96.36, 90°	42.30, 46.54, 91.99 Å; 95.65, 100.11, 90.08°	101.7, 118.3, 122.5 Å; 90, 90, 90°
Resolution range (Å)	50–2.75 (3.15–2.75)	50–2.50 (2.56–2.50)	50–1.73 (1.77–1.73)	50–1.50 (1.59–1.50)	50–2.55 (2.62–2.55)
⟨I/σI⟩	10.3 (1.6)	5.5 (0.6)	11.8 (1.0)	6.9 (0.5)	11.6 (0.5)
Completeness (%)	Spherical 70.0 (18.0) [ellipsoidal 99.6 (66.7)]	91.7 (91.3)	99.1 (98.7)	89.7 (89.4)	99.8 (99.8)
Redundancy	5.6 (6.3)	3.8 (3.8)	5.6 (6.3)	2.7 (2.7)	7.5 (7.4)
*R*_meas_ (%)	14.2 (136.3)	20.5 (291.4)	8.0 (225.6)	9.1 (236.8)	11.2 (349.6)
CC_½_ (%)	99.6 (58.0)	99.2 (33.5)	99.8 (35.9)	99.7 (22.4)	99.9 (33.5)
Structure refinement					
*R*_cryst_/*R*_free_ (%)	28.9/32.3	25.4/29.9	19.0/21.5	17.9/22.9	28.0/30.3
RMSD bond lengths (Å)/ angles (°)	0.002/0.6	0.004/0.7	0.011/1.1	0.011/1.0	0.005/0.7
Ramachandran favoured/outliers (%)	89.5/2.1	95.1/0.8	97.2/0.6	98.1/0.0	94.4/0.9
MolProbity score/percentile	2.86/68th	2.22/89th	1.74/77th	1.24/96th	2.48/80th
Protein chains in asymmetric unit	2	8	2	4	8
PDB entry	9ERT	9ERU	9ERW	9ETL	9ETJ

*Note*: The values in parentheses correspond to the highest‐resolution shell.

### 2.17 Small‐angle X‐ray scattering

SAXS data were collected on the beamlines SWING (Thureau et al., 2021) at the SOLEIL synchrotron (Gif‐sur‐Yvette, France) and BM29 (Pernot et al., 2013) at the ESRF (Grenoble, France). To measure solution scattering of CNPase‐Nb complexes, Nbs were mixed with the CNPase catalytic domain in 1.3‐fold molar excess, and data were collected using an SEC‐SAXS setup. The column used was Bio‐SEC‐3130 Å 4.6/300 (Agilent Technologies; RRID:SCR_013575). All samples were run in 20 mM HEPES, 150 mM NaCl, 0.5 mM TCEP and pH 7.5. Data were processed using FOXTROT (SOLEIL synchrotron) and ATSAS (RRID:SCR_015648; Manalastas‐Cantos et al., 2021). Frame selection and buffer subtraction were carried out in CHROMIXS (Panjkovich & Svergun, 2018), primary analysis in PRIMUS (Konarev et al., 2003), and distance distribution analysis in GNOM (Svergun, 1992). Ab initio models were created using DAMMIN (Svergun, 1999) and GASBOR (Svergun et al., 2001) and multi‐phase models in MONSA (Svergun, 1999). Theoretical scattering curves for crystal structures were calculated using CRYSOL (Svergun et al., 1995).

### 2.18 Activity assay in vitro

The 3′‐phosphodiesterase activity was measured as described (Myllykoski et al., 2013; Sogin, 1976), using 2′,3′‐cyclic NADP^+^ (Biolog Life Science Institute, Bremen, Germany; Cat. No. N050) as the substrate in a coupled assay. Activity was measured in 100 mM Bis‐Tris pH 6.0, 10 mM MgCl_2_ in the presence of 1 U glucose‐6‐phosphate dehydrogenase, 10 mM glucose‐6‐phosphate, and 0–2 mM 2′,3′‐cNADP^+^ at +25°C in 96‐well plates in a Spark 20 M multimode plate reader (Tecan Life Sciences, Switzerland; RRID:SCR_021897). To initiate the reaction, 2× solutions of serially diluted substrate were mixed with a 2× solution of the reaction buffer, CNPase, G6P, and G6P‐dehydrogenase, and the formation of NADPH was monitored for 20 min. Substrate concentrations were determined via absorbance at 259 nm (*ε* = 18 mM^−1^ cm^−1^). Initial velocities were plotted as a function of substrate concentration and kinetic parameters obtained by fitting with the non‐linear Michaelis–Menten function in GraphPad Prism (RRID:SCR_002798).

## 3 RESULTS

### 3.1 Purification and characterisation of recombinant anti‐CNPase nanobodies

**FIGURE 1:**
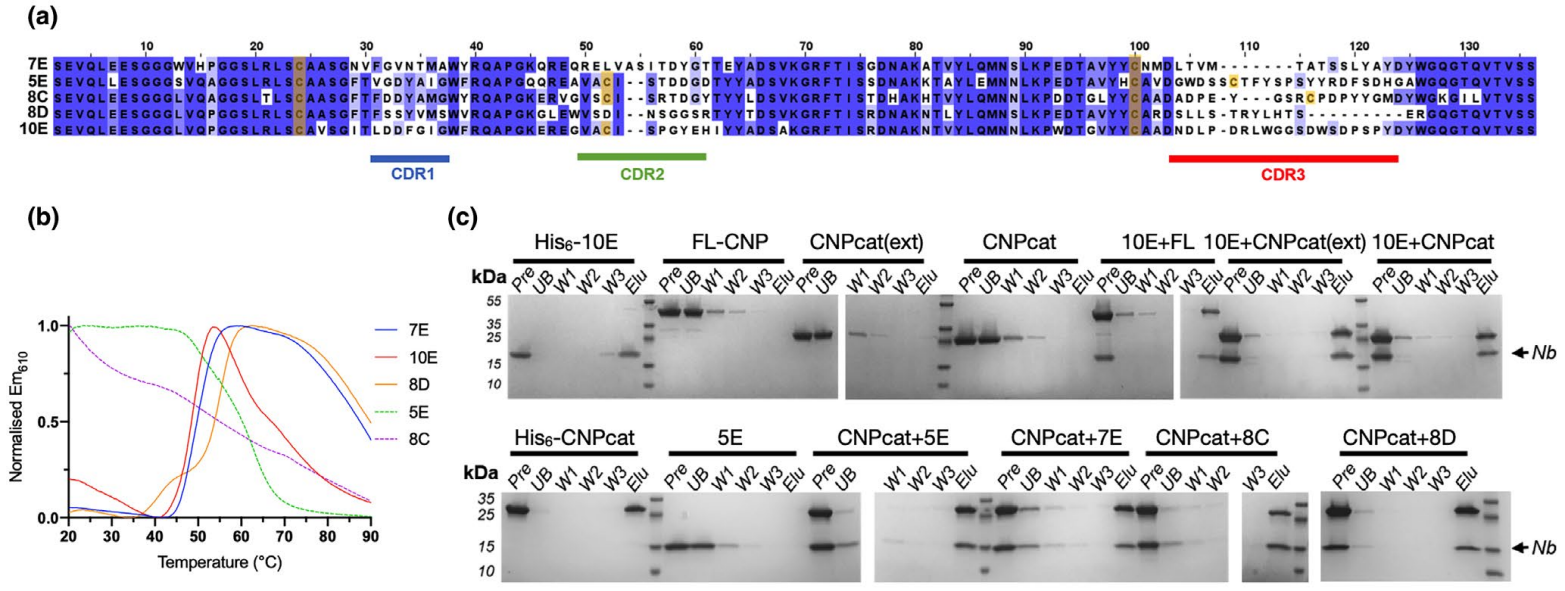
Characteristics of anti‐CNPase nanobodies. (a) Sequence alignment of the five NbCNPs. Cys residues are indicated in yellow. (b) DSF analysis of Nb stability. (c) Pulldown assay indicates all Nbs bind to the CNPase catalytic domain. FL‐CNP, full‐length CNPase; CNPcat, CNPase catalytic domain; CNPcat(ext), CNPase catalytic domain with C‐terminal tail. Pre, sample loaded onto affinity matrix; UB, unbound fraction; W1‐3, wash fractions; Elu, eluted fraction. The full SDS‐PAGE gels are shown in Figure S1.

Five anti‐CNPase Nbs—5E, 7E, 8C, 8D, and 10E—were raised against CNPase via alpaca immunisation with rat synaptosomes (Queiroz Zetune Villa Real et al., 2023). Alignment of the NbCNP sequences highlights the large size of their complementarity‐determining region 3 (CDR3) loops (Figure 1a). Furthermore, cysteine residues are present in the CDR2s of NbCNP 5E, 8C, and 10E and the CDR3 of 5E and 8C. All NbCNPs could be expressed in *E. coli* and purified for structural studies of CNPase‐Nb complexes. DSF was used to assess NbCNP thermal stability (Figure 1b). NbCNP 7E and 10E had *T*_m_ of +50°C, and NbCNP 8D was slightly more thermostable, with a *T*_m_ of +55°C. Unfolding transitions were not observed for Nbs 5E and 8C, and the initial fluorescence signal resembled that of an unfolded state. As these Nbs showed binding to CNPase, this could indicate the presence of hydrophobic regions, perhaps in the CDRs, binding the assay dye in the folded state. Pull‐down assays indicated that all five NbCNPs bound to the C‐terminal phosphodiesterase domain of CNPase (Figure 1c).

Mass spectrometry was used to confirm the identity of the recombinant NbCNPs (Table S1). The observed masses were used to estimate the oxidation state of cysteines. Apparently, the conserved disulphide of both NbCNP‐7E and 10E was reduced; its formation is, therefore, not essential for folding. The conserved central disulphide and the additional cysteines in the CDR2 and CDR3 of NbCNPs 5E and 8C were oxidised upon folding. The mass of 8C indicated two disulphides, and 5E gave two peaks of masses corresponding to one and two disulphides. These data suggest that Cys residues in the CDR loops of NbCNPs 5E and 8C may stabilise the paratope conformation through disulphide bridges.

### 3.2 Nanobodies bind CNPase at high affinity and increase its thermal stability

**FIGURE 2:**
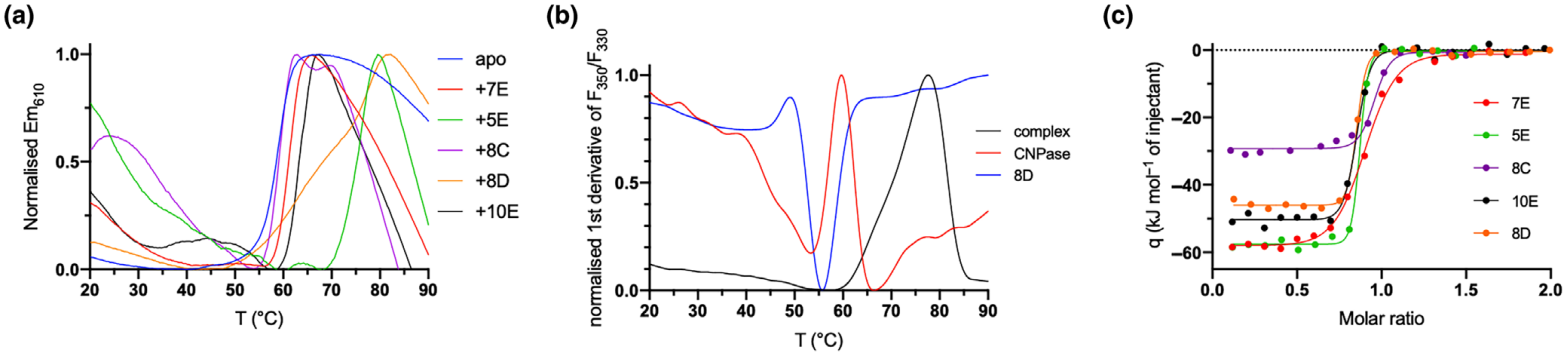
NbCNP binding to full‐length mCNPase. (a) Interaction probed via a DSF thermal shift assay. (b) NanoDSF experiment for NbCNP 8D. (c) ITC titration of Nbs into CNPase.

The effects of all five NbCNPs on the thermal stability of full‐length mouse CNPase were studied using DSF (Figure 2a; Table 3). All Nbs showed thermal stabilisation of CNPase, with the strongest stabilisation observed for NbCNPs 5E and 8D. The reproducibility of the observation was shown by an additional label‐free nanoDSF experiment for the CNPase‐8D complex (Figure 2b; Table 3).

**TABLE 3:** Thermal stabilisation of full‐length CNPase upon NbCNP binding.

Sample	*T*_m_ (°C)	∆*T*_m_ (°C)
CNPase		
DSF	59.2 ± 0.1	‐
nanoDSF	59.7 ± 0.03	‐
+7E	61.3 ± 0.3	+2.1
+5E	76.2 ± 0.3	+17.0
+8C	68.4 ± 0.5	+9.2
+8D		
DSF	77.2 ± 0.3	+18.0
nanoDSF	77.4 ± 0.1	+17.7
+10E	63.4 ± 0.6	+4.2

*Note*: *T*_m_ values were determined using DSF (*N* = 3). NbCNP 8D was additionally studied using label‐free nanoDSF.

The binding affinity was examined using ITC (Figure 2c; Table 4). Most of the NbCNPs bound with a *K*_d_ of 0.5–15 nM; NbCNP 7E showed affinity decreased by an order of magnitude. The contributions of the enthalpy and entropy terms varied, suggesting diverse modes of enthalpy‐driven binding.

**TABLE 4:** Thermodynamic parameters of NbCNP binding to CNPase catalytic domain.

Nb	Number of sites	*K*_d_ (nM)	∆H (kJ mol^−1^)	‐T∆S (kJ mol^−1^)	∆G (kJ mol^−1^)
5E	0.820 ± 0.003	2.2 ± 0.8	−57.70 ± 0.42	+8.27	−49.43
7E	0.863 ± 0.005	83.3 ± 9.3	−59.25 ± 0.52	+18.78	−40.47
8C	0.914 ± 0.007	16.6 ± 5.5	−29.59 ± 0.52	−14.82	−44.41
10E	0.804 ± 0.005	9.8 ± 2.9	−50.63 ± 0.64	+4.89	−45.74
MaBP‐10E	0.836 ± 0.004	13.4 ± 3.1	−75.60 ± 0.79	+31.01	−44.90
MaBP‐8D	0.837 ± 0.004	0.2 ± 0.4	−45.90 ± 0.49	−9.43	−55.35
8D	0.800 ± 0.002	2.5 ± 1.6	−46.11 ± 0.36	−3.02	−49.13

*Note*: Error margins are from data fits for single experiments.

Initially, insufficient amounts of pure NbCNP‐8D could be obtained for the ITC assay, and the NbCNP 8D fusion with the intact His_6_‐MaBP (maltose‐binding protein) was used for the ITC experiment. As a control, the binding of NbCNP 10E with and without the MaBP tag was studied. The MaBP‐8D fusion showed the highest binding affinity of all, with a *K*_d_ <1 nM. MaBP‐10E bound with a *K*_d_ similar to NbCNP 10E with the fusion tag removed, and therefore, the *K*_d_ measured for MaBP‐8D likely approximates to that of the untagged Nb, and MaBP has no effect on binding. An experiment carried out on purified Nb 8D confirmed that 5E and 8D were the NbCNPs with the highest affinity towards the mCNPase catalytic domain; in fact, the affinities are so high that they cannot be accurately determined using ITC under the current conditions. Altogether, ITC demonstrated that all NbCNPs bind the catalytic domain of mCNPase with high affinity, with varying degrees of enthalpy‐entropy compensation. However, the Nbs may additionally bind the N‐terminal PNK‐like domain in the full‐length protein, which will be a subject for future experiments. The result additionally shows that a fusion of NbCNPs with a larger partner, MaBP, has no effect on binding affinity.

### 3.3 Structure of CNPase‐Nb complexes

**FIGURE 3:**
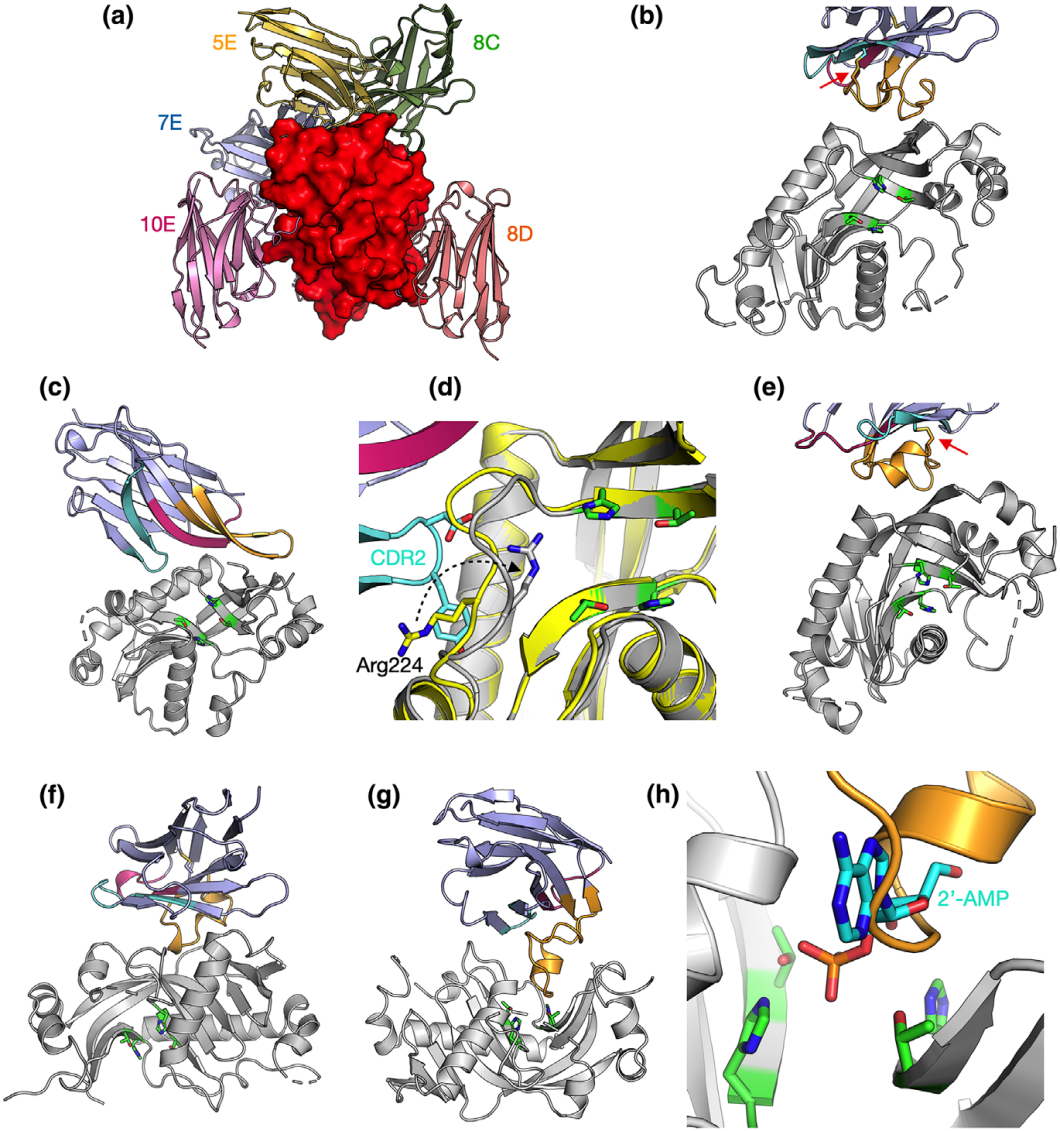
The five anti‐CNPase Nbs all have different epitopes. (a) The individual crystal structures have been superimposed based on the CNPase catalytic domain (red surface). Individual structures: (b) 5E. (c) 7E. (d) Zoom into the conformational change of Arg224 (arrow) near the active site, induced by the CDR2 loop. (e) 8C. (f) 8D. (g) 10E. (h) Zoom into the active site in the 10E complex, showing CDR3 overlapping with the binding mode of the reaction product 2′‐AMP (cyan; Myllykoski, Raasakka, et al., 2012). The CDR loops (CDR1, magenta; CDR2, cyan; CDR3, orange) and catalytic residues (green; two HxTx motifs) are highlighted in panels b–h. The disulphide bridge between CDR2 and CDR3 in NbCNPs 5E and 8C is indicated with a red arrow (panels b, e).

The structures of the CNPase catalytic domain in complex with all five NbCNPs were determined by X‐ray crystallography (Figure 3; Table 2). In all cases, the bound Nb could be almost completely built into the electron density, but flexibility in some solvent‐exposed loops of CNPase resulted in poor density. Each NbCNP has a different epitope on the CNPase catalytic domain (Figure 3a). NbCNPs 8C and 8D bind the furthest away from the active site (Figure 3a,e,f).

In NbCNPs 5E and 8C, the CDR3 loop is linked to the central Nb fold via an extra disulphide bond between CDR2 and CDR3 (Figure 3b,e), as supported by the mass spectrometry data (Table S1). Accordingly, the CDR3 forms a large flat paratope, predominantly binding a hydrophobic surface patch on CNPase. Both 5E and 8C interactions are primarily mediated by aromatic residues concentrated in the CDR3.

In contrast, 7E and 10E lack a cysteine in the CDR3, which extends away from the central Nb fold to bind the epitope. In 7E, the CDR1 and CDR2 bound to CNPase in proximity to the active site altering the conformation of a flexible loop and Arg224, while the elongated CDR3 loop formed β‐sheet‐like interactions with the CNPase C‐terminal region, which lies close to the N terminus of the catalytic domain (Figure 3c,d). A large portion of the 7E CDR3 loop does not bind CNPase, which can explain its lower affinity and suggests that the loop might bind to the interdomain interface in full‐length CNPase.

The elongated CDR3 of NbCNP‐10E extends into the CNPase active site and overlaps with the substrate/product binding site (Figure 3g,h). Moreover, the CDR1 and CDR2 of 10E bound a second CNPase molecule in the crystal, apparently fixing the protein in a homodimeric state in the crystal. Possible dimerisation was confirmed using PISA, and the calculated ∆G^int^ of −64.02 kJ mol^−1^ suggested a stable assembly. This putative dimer interface formed between hydrophobic surface patches of CNPase and buried 930 Å^2^ of the solvent‐accessible surface of each monomer. This dimerisation, also suggested by the SAXS experiments below, could represent an oligomeric state of full‐length CNPase in vivo since it was picked up by NbCNP 10E. In addition, it may be linked to the inhibitory mode of NbCNP 10E.

### 3.4 Solution structure of CNPase catalytic domain‐nanobody complexes

**FIGURE 4:**
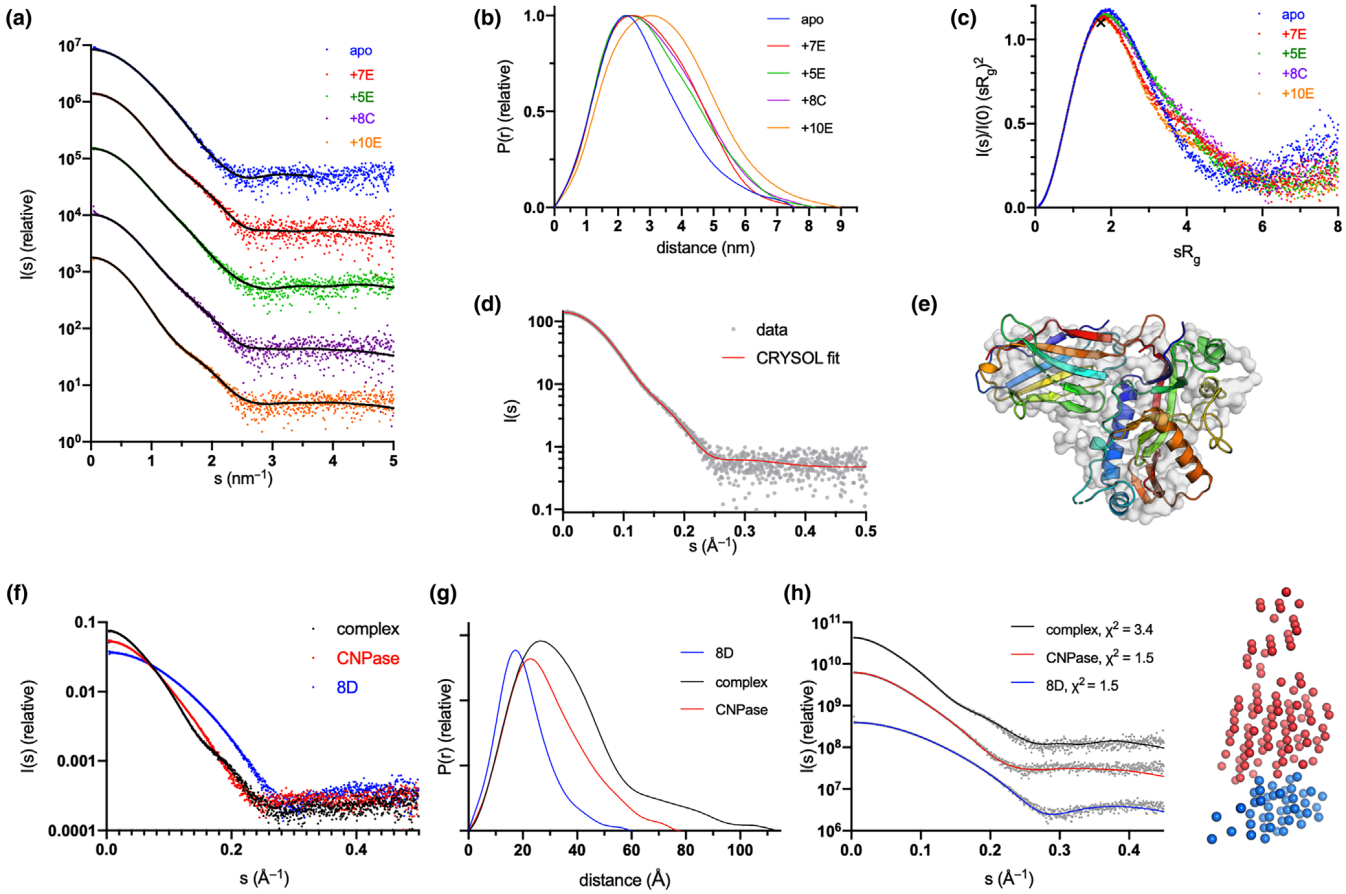
Conformation of CNPase‐nanobody complexes in solution. (a) Scattering data, with fits of chain‐like ab initio models as lines. (b) Distance distribution. (c) Dimensionless Kratky plot. The position of the cross corresponds to a theoretical maximum for a globular particle. The colouring scheme is identical in panels (a–c). (d) Fit of the CNPase‐NbCNP 7E crystal structure to the raw SAXS data. (e) Superposition of the chain‐like SAXS model of CNPase‐NbCNP 7E (surface) and the crystal structure (cartoon). Fits for all complexes to the SAXS data are shown in Figure S2. (f) Scattering data for the NbCNP 8D complex. (g) Distance distribution for NbCNP 8D. The colouring scheme is identical in panels (f–g). (h) Fit of the multi‐phase MONSA model to all datasets simultaneously. The model of the complex is shown on the right (red—CNPase, blue—NbCNP 8D). The NbCNP 8D complex data are shown separately due to the different expression constructs used for nanobody purification and for comparing all components in the same experiment.

To observe and validate the complexes in solution, we conducted synchrotron SEC‐SAXS experiments on the CNPase catalytic domain and the NbCNPs (Figure 4; Table 5). All CNPase catalytic domain‐Nb complexes eluted as single peaks, suggesting one dominant oligomeric state. All complexes were 1:1 heterodimers and fit well to the SAXS data and closely resembled ab initio chain‐like models (Figure 4; Figure S2), except for the NbCNP10E, for which the complex had a slightly higher apparent molecular weight. This may indicate Nb‐induced dimerisation of the CNPase catalytic domain or vice versa, which could relate to the mode of inhibition of CNPase catalysis by 10E. The assembly from the crystal that fit the SAXS data best contained 2 molecules of CNPase and one 10E molecule. For the case of NbCNP 10E, as well as full‐length CNPase, additional studies will be required to conclude on detailed structural properties of the complex.

**TABLE 5:** Size and shape parameters of CNPase‐NbCNP complexes derived from SAXS data.

Sample	CNPase	+5E	+7E	+8C	+8D	+10E	+MaBP‐8D
*R*_g_ (nm)	2.18	2.38	2.36	2.41	2.82	2.65	3.77
*D*_max_ (nm)	7.5	8.2	7.6	8.0	11.0	9.0	16.1
*Q*_p_ MW (kDa)	24.3	34.1	33.2	33.3	46.0	50.9	85.3
Theoretical MW 1:1 complex (kDa)	24.1	38.3	37.9	38.1	39.0	38.5	81.3
Oligomeric state	Monomer	1:1	1:1	1:1	1:1	1:2 or 2:1	1:1
*χ* ^2^ of crystal structure	21.3	2.2	1.2	1.4	472.8	238.1	‐

*Note*: The catalytic domain of mCNPase was used in the experiments. The fits of the crystal structures (from CRYSOL) to the solution SAXS data indicate that CNPase alone is more extended in solution than in the crystal, the CNPase‐8D complex in solution has an extended tail, and the CNPase‐10E complex stoichiometry differs from 1:1 (see text and Figure S2 for details).

The varying *R*_g_ and *D*_max_ among the complexes suggested different binding modes. The NbCNP 8D complex with CNPase shows a higher *D*_max_ due to the different construct used to produce the Nb for these experiments, with a short, extended tail. The NbCNP 8D SAXS data alone showed the same elongated feature (*R*_g_ 1.57 nm, *D*_max_ 6.0 nm), larger than expected for a compact Nb single domain. This explains why the crystal structure provides a poor fit to the experimental SAXS data in this case (Table 5; Figure S2). As an alternative modelling protocol, a two‐phase model for the CNPase‐8D complex was built, using data for both components and the complex simultaneously (Figure 4f–h). The model fits both the individual components and the complex, supporting a 1:1 complex between CNPase and NbCNP 8D. The slightly poor fit of the crystal structure to the raw SAXS data in the case of NbCNP 8D is likely explained by disordered regions not ordered in the crystal, but present in solution. The stoichiometry was additionally confirmed by an experiment using the MaBP‐NbCNP 8D fusion bound to CNPase (Table 5).

### 3.5 CNPase activity

**FIGURE 5:**
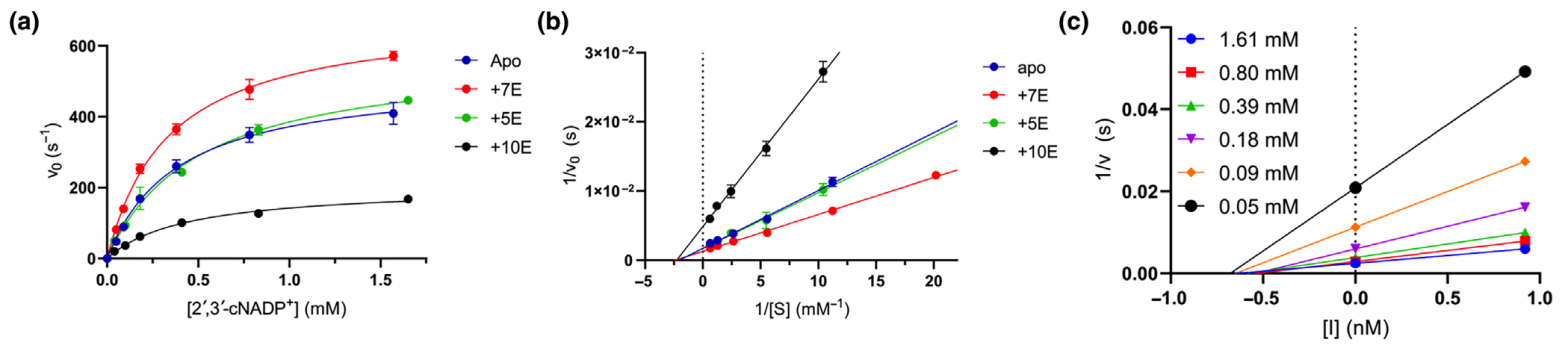
CNPase catalytic activity in the presence of selected nanobodies. (a) Initial rates of 2′,3′‐cNADP^+^ hydrolysis. (b) Lineweaver‐Burk plot indicates non‐competitive inhibition by NbCNP 10E. (c) Non‐competitive Dixon plot in the presence of NbCNP 10E. [I] denotes the concentration of 10E, and the *x*‐axis intercept gives apparent −*K*_i_. Each curve represents a different substrate concentration (panel a).

To explore the effect of Nb binding on CNPase function, the enzymatic activity of the full‐length mCNPase was measured in complex with NbCNP‐5E, −7E, and −10E (Figure 5; Table 6). Full‐length mCNPase‐nanobody complexes purified by SEC were used in the experiment. NbCNP‐5E had little to no effect on CNPase activity (Table 6). However, with NbCNP‐7E, a slight increase in *k*_cat_ was apparent, indicating activation of CNPase upon 7E binding close to the active site. NbCNP‐10E was a non‐competitive inhibitor of CNPase (Figure 5a,b), facilitating a considerable reduction in *k*_cat_, with an unchanged *K*_M_. The inhibition was potent, with an apparent *K*_i_ of 0.6 nM (Figure 5c; Table 6), in line with the high affinity of the nanobody towards CNPase. Non‐competitive inhibition results from inhibitor binding to both the free enzyme and the enzyme‐substrate complex; confirmation of this inhibition mode will require experiments with a wider range of nanobody concentrations in the future. More comprehensive kinetics experiments will need to be carried out to fully unravel the effects of the Nbs on CNPase activity.

**TABLE 6:** Kinetic parameters of full‐length mCNPase in the presence of selected nanobodies.

Bound state	*k*_cat_ (s^−1^)	*K*_M_ (μM)	*k*_cat_/*K*_M_ (M^−1^ s^−1^)	Effect	*K*_i_ (pM)
Apo	578.7 ± 52.0	482.9	1.20 × 10^6^	N/A	N/A
+5E	596.0 ± 92.9	481.9	1.24 × 10^6^	Activation	N/A
+7E	773.4 ± 43.8	411.9	1.88 × 10^6^	None	N/A
+10E	205.6 ± 16.1	438.9	4.69 × 10^5^	Non‐competitive inhibition	603.5 ± 50.7

*Note*: *k*_cat_ and *K*_M_ were obtained from the Lineweaver‐Burk plot and the apparent *K*_i_ from the Dixon plot (Figure 5b,c). The errors represent standard deviation from the curve fitting of the shown experiments.

Abbreviation: N/A, not applicable.

### 3.6 Nanobody specificity in imaging applications

NbCNP 5E and 8D were selected for further validation because of their strong affinity towards the catalytic domain of mCNPase. NbCNP 10E was chosen because of its specific binding into the active site. NbCNPs 5E, 8D, and 10E with an ectopic cysteine at the C‐terminus were prepared, and site‐specific conjugation was performed using maleimide‐functionalised fluorophores (Oleksiievets et al., 2022; Queiroz Zetune Villa Real et al., 2023).

**FIGURE 6:**
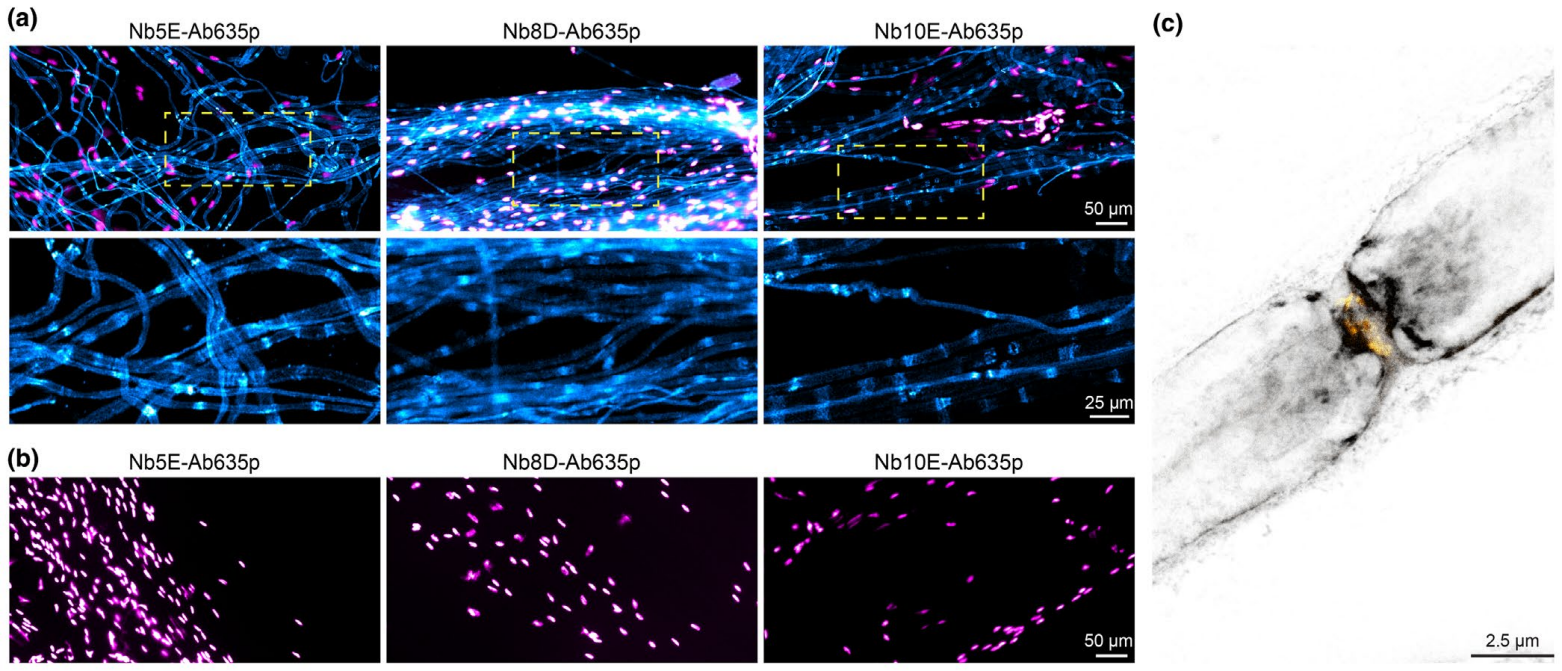
Specific staining of mCNPase using fluorescently labelled nanobodies. (a) Epifluorescence images of teased sciatic nerves from wild‐type mice stained with Nb5E, Nb8D, and Nb10E displayed in cyan, nuclei stained with DAPI displayed in magenta. (b) Teased sciatic nerves from mice lacking CNPase display no staining (cyan) and nuclei in magenta. Images were acquired with the same acquisition settings and are equally scaled for display and direct comparison. (c) 2‐Colour 2D STED image using Nb8D‐Ab635p (grey) and voltage‐gated sodium channels (Nav1.6; yellow).

For a specificity test, we prepared teased sciatic nerves from wild‐type mice and mice lacking CNPase (Lappe‐Siefke et al., 2003) and stained them with the fluorescently labelled NbCNPs. We obtained specific signals on the wild‐type nerves with all three selected Nbs (Figure 6a), while no signal was observed from the CNPase‐deficient tissue (Figure 6b), indicating the specificity of the signal. All Nbs detected mCNPase throughout the non‐compacted regions of the myelin sheath; a stronger signal can be observed on the paranodes where the nanobodies have better access to target CNPase.

We then performed 2‐colour STED microscopy using Nb8D‐Ab635p and antibodies against voltage‐gated sodium channels (Nav1.6) on the node of Ranvier, confirming that the nanobody signal is enhanced on the paranodes (Figure 6c).

**FIGURE 7:**
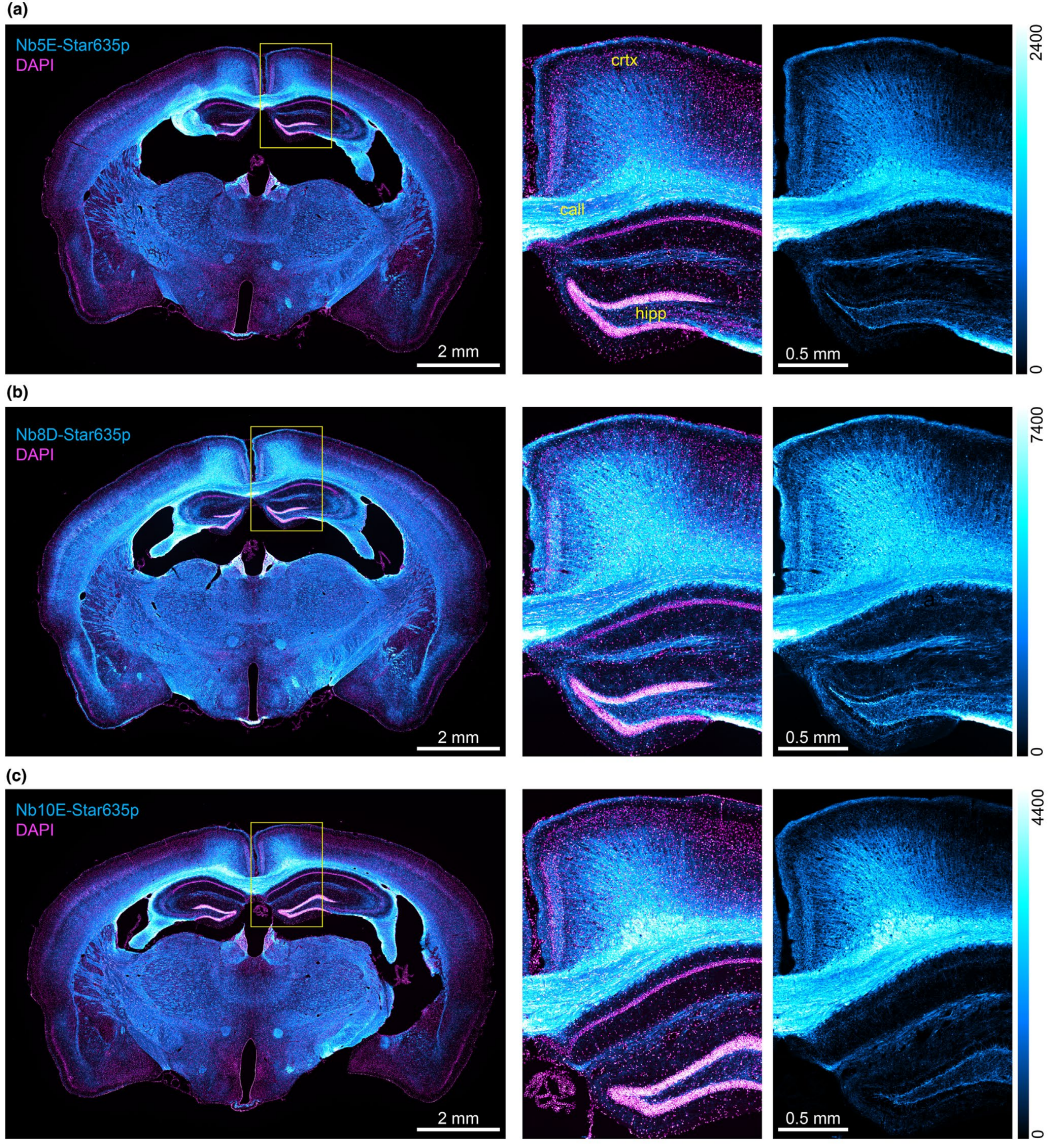
Immunohistochemistry on mouse brain tissue sections. Epifluorescence images of coronal mouse brain sections. In cyan, the stainings of (a) NbCNP 5E, (b) NbCNP 8D, and (c) NbCNP 10E. Nuclei stained with DAPI in magenta. Yellow rectangles show the zoomed region, including the hippocampus (hipp), corpus callosum (call), and cortex (crtx) as an overlay (CNPase + nuclei) and single colour (CNPase). Images were acquired with the same conditions. Thus, the intensity scale (in arbitrary units) for the cyan look‐up table is displayed to the right of each panel as a reference and for direct brightness comparison.

Finally, we used the NbCNPs for a challenging test by performing immunohistochemistry on mouse brain tissue sections (Figure 7; Figure S3). We observed highly efficient staining of myelinated tracts in the brain, and there was virtually no background staining in regions where myelin was not expected. In addition, we could observe the different affinities of NbCNPs by comparing the fluorescence intensities, for which NbCNP 8D was significantly brighter, providing an excellent signal‐to‐noise ratio; we looked at higher magnification in some of the regions (Figure S3). The crystal structures indicate that NbCNPs 5E, 8D, and 10E should not sterically interfere with the binding of one another, as they bind onto different faces of CNPase (Figure 3a). Based on this information, we tested if we could obtain a signal amplification when using these three Nbs in an oligomeric cocktail. Our observation suggests that combining the three Nbs resulted in a 2.8 ± 0.3 ‐fold increase in the specific signal (Figure S4).

### 3.7 Expression of NbCNPs as intrabodies (iNbs)

**FIGURE 8:**
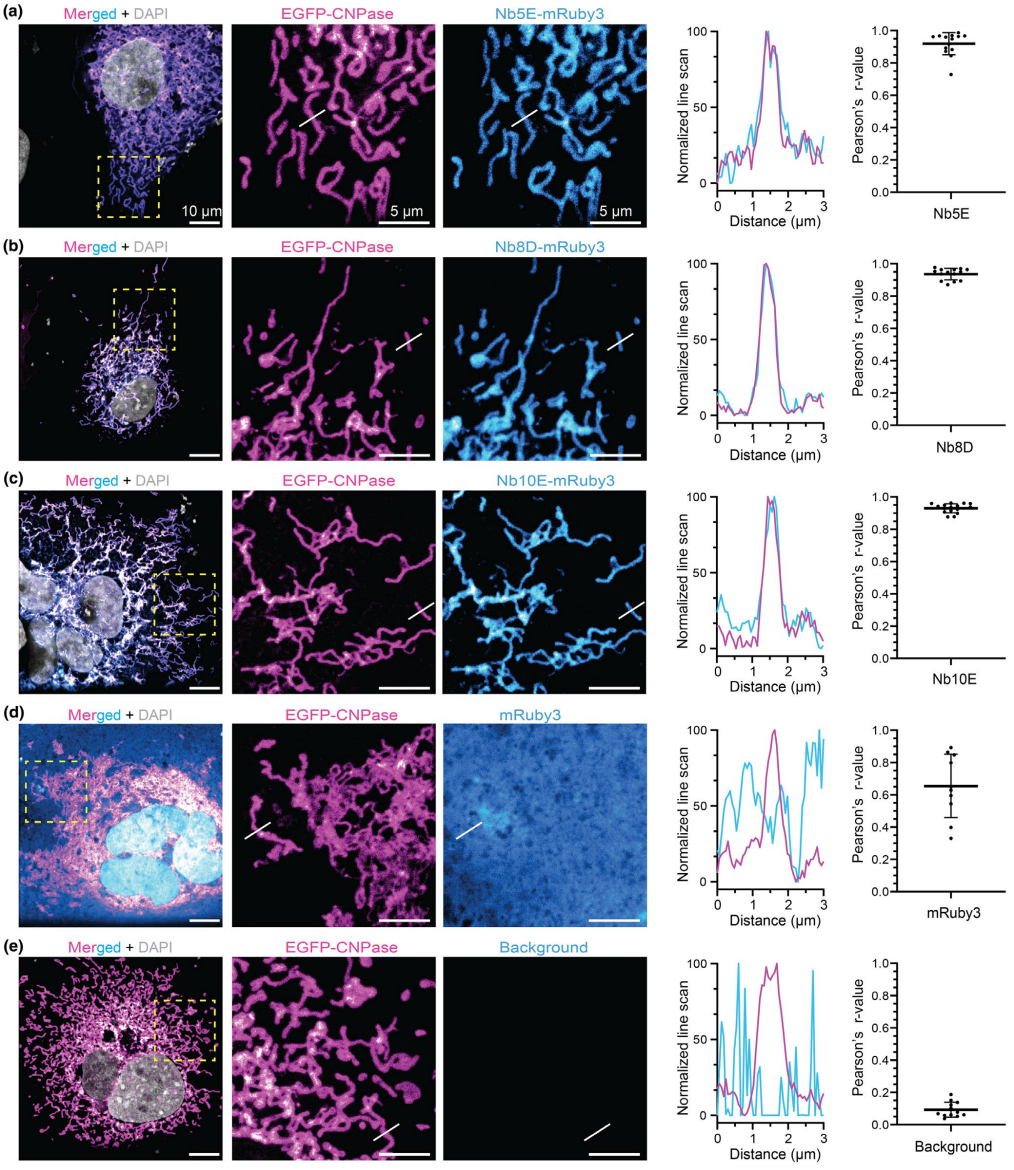
Nanobodies as intrabodies in COS‐7 cells. Confocal images were acquired after COS‐7 cells were co‐transfected with TOM70‐EGFP‐CNPase and an iNb fused to mRuby3. (a) Merged signals in a wide field of view, followed by zoomed regions (yellow square) displaying the mitochondria structures by TOM70‐EGFP‐CNPase (magenta) and the signal from mRuby3 fused to iNbCNP 5E. White lines denote the position where the line‐intensity profile (line‐profile) is shown in the graph. Pearson's correlation between the mitochondrial EGFP signal (magenta) and the mRuby3 signal from the iNb (cyan) was calculated from *N* = 9 cells. The graph represents the mean ± standard deviation (SD). The same analysis was performed for iNbCNP 8D (b), iNbCNP 10E (c), and the controls with cytosolic mRuby3 not fused to any iNb (d), or no iNb transfected (e). This last control rules out potential signal bleed‐through from EGFP into the mRuby3 channel.

As a means to validate the selected NbCNPs as intrabodies, the NbCNPs 5E, 8D, and 10E fused to mRuby3 were co‐expressed with TOM70‐EGFP‐CNPase (catalytic domain) in COS‐7 cells. These Nbs were selected due to their high affinity and effect on CNPase activity. In this system, an EGFP‐tagged CNPase is anchored to the mitochondrial outer membrane (TOM70). If Nbs are functional as iNbs in the reducing cytoplasmic environment of living cells, we expected a co‐localisation between EGFP and mRuby3 signals. All three tested iNbs showed strong colocalisation (Pearson's correlation) with the CNPase anchored to mitochondria. In contrast, the expression of mRuby3 alone as negative control shows poor specific co‐localisation, demonstrating that these NbCNPs can be used as iNbs against CNPase (Figure 8). Specially interesting for future applications is the NbCNP 10E, which also inhibits CNPase catalytic activity (Figure 5).

**FIGURE 9:**
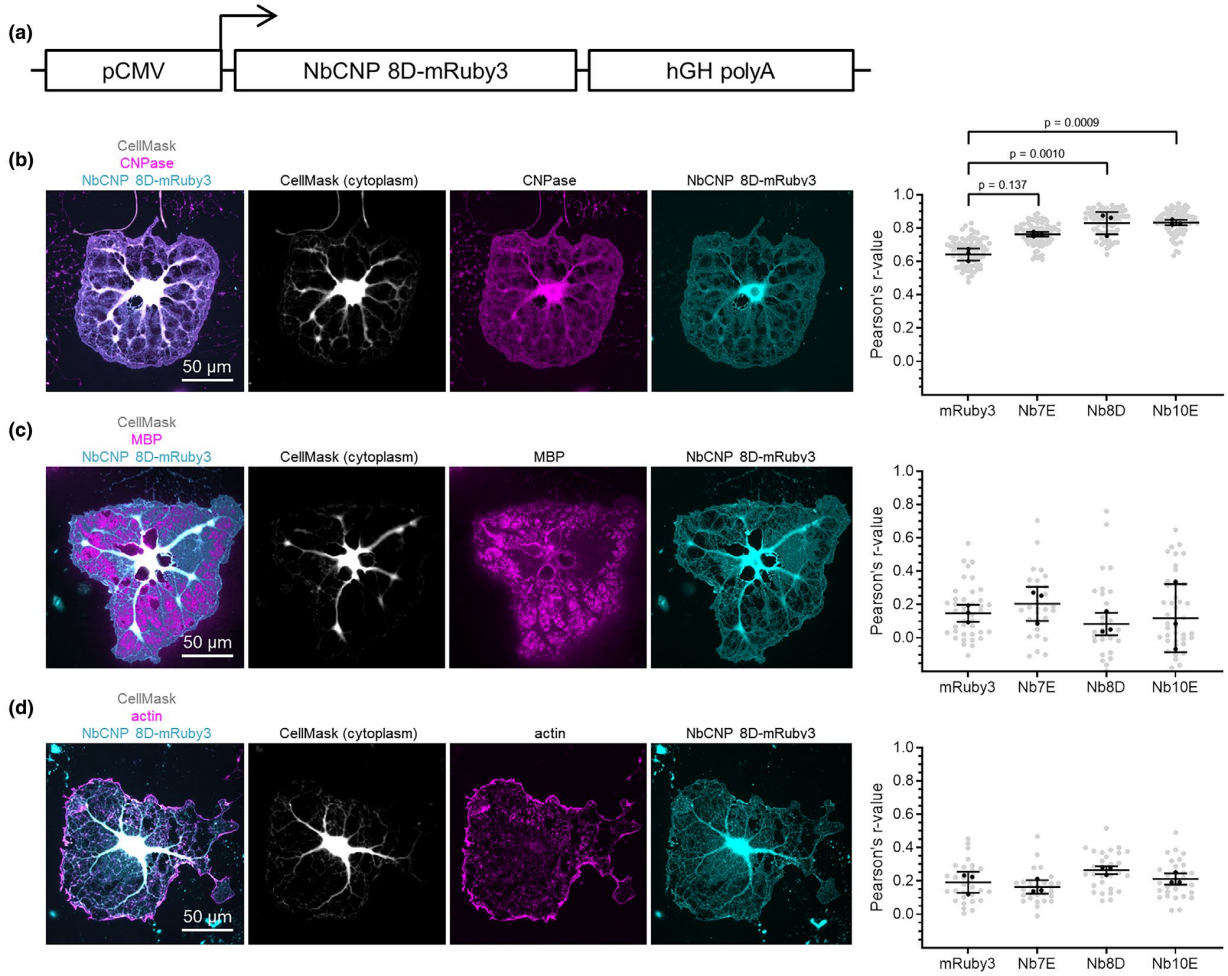
Expression of NbCNPs as intrabodies in cultured oligodendrocytes. Shown is the experiment with iNbCNP 8D. Experiments with the other iNbs are shown in Figure S5. (a) The iNbCNP 8D construct architecture, with fused mRuby3, used in transfection. NbCNP‐8D‐mRuby3‐expressing cells were stained for either CNPase (b), MBP (c), or filamentous actin (d). Fluorescence for the labelled iNbCNP was directly recorded. Graphs to the right show Pearson's correlation coefficients as mean ± SD, which were calculated between mRuby3 and the given staining for each panel (b, Nb to CNPase; c, Nb to MBP; d, Nb to F‐actin). Means of individual biological replicates (*n* = 3) are plotted as black dots and values from individual cells as grey dots (see Table S2 for details). Statistical significances (*p*‐values) were calculated from the means of biological replicates using one‐way ANOVA followed by Dunnett's multiple comparisons test (Table S3).

All five NbCNPs were then tested for expression as iNbs in primary cultured oligodendrocytes (Figure 9; Figure S5), which is the cell type in which CNPase is endogenously expressed. The NbCNPs 7E, 8D and 10E, but not 5E and 8C, expressed in cultured oligodendrocytes as iNbs and co‐localised with CNPase immunostaining in cytoplasmic channels, as well as in cytoplasmic patches among compaction zones, at day 5 of differentiation. As expected, there was little to no co‐localisation with myelin basic protein (MBP) on average (Aggarwal et al., 2011). Since CNPase interaction with actin filaments has been shown (Snaidero et al., 2017), actin was stained with phalloidin. Some potential iNbCNP co‐localisation was detected with actin, although most actin at day 5 of differentiation is present as a rim at the edges of the cell, resulting in a low Pearson's correlation on average.

The iNbCNP‐mRuby3 fusions 5E and 8C did not produce fluorescence in the oligodendrocytes; hence, there was either no expression or the protein aggregated. Interestingly, these are the two Nbs with an extra disulphide bridge linking CDR2 and CDR3, which may reflect a folding issue in the reducing cellular environment; the putatively different redox environments of COS‐7 cells and oligodendrocytes likely explain some of the differences between experiments. In addition, NbCNP 10E decreased CNPase staining by the conventional antibody (Figure S5d,e). 10E may bind the same epitope as the antibody used for immunostaining in the experiment, or oligodendrocytes might remove/degrade non‐functional CNPase when inhibited by iNbCNP 10E. Thus, NbCNPs can be expressed as intrabodies inside cells including oligodendrocytes, where they may enable future studies of the cellular roles of CNPase in myelin formation or disease.

## 4 DISCUSSION

### 4.1 CNPase and myelin

Myelin serves as an electrical insulator, preventing leakage of electrical signals along the axon. This allows for rapid and efficient saltatory conduction, in which the electrical signal jumps from node to node, enabling faster transmission of information compared to unmyelinated axons. Myelin also provides structural support to axons, protecting them from damage and degeneration. Additionally, myelin plays a role in nutrient transport and waste removal from axons. Understanding the biology of myelin and the mechanisms underlying myelin disorders will be crucial for developing effective treatments for demyelinating disorders in the future.

Due to its high abundance in myelinating glia, CNPase is a commonly used marker of myelin, being localised in the non‐compacted subcompartments (Trapp et al., 1988). The molecular function of CNPase is not fully understood (Raasakka & Kursula, 2014), and a high‐resolution structure of full‐length CNPase has not been experimentally solved; crystal structures exist for only the phosphodiesterase domain in complex with different active‐site ligands (Myllykoski et al., 2013; Myllykoski, Raasakka, et al., 2012; Raasakka et al., 2015). To characterise different aspects of CNPase function, solve the high‐resolution structure of full‐length CNPase, and improve the use of CNPase as an imaging and diagnostic tool for both the CNS and PNS, new approaches are required. To reach these goals, we developed a set of high‐affinity Nb binders against the phosphodiesterase domain of CNPase.

### 4.2 Nb screening and production

The anti‐CNPase Nbs presented here resulted from a fortuitous immune reaction against myelin protein contaminants in a rat brain synaptosome preparation that was used for alpaca immunisation. Since the major myelin proteins, including CNPase, are abundant in nerve tissue, this was not fully unexpected. The result suggests high antigenicity of CNPase, given that it was an impurity in the preparation; interestingly, all epitopes for the Nbs lie on the C‐terminal catalytic domain, each with its distinct epitope.

With some modifications in the protocols, all NbCNPs could be produced recombinantly on a large scale, and some of the unexpected yield and solubility issues might have been caused by the extra Cys residues present in the CDR loops. For two of the Nbs, these form a disulphide bridge between CDR2 and CDR3, stabilising the CDR conformation. Such cysteine residues in exposed loops are likely reactive and can cause problems during recombinant production on a large scale.

The high‐quality recombinant anti‐CNPase nanobodies can be additionally modified and optimised for further applications, which—as shown here—can include structural biology, imaging, and functional intervention. As a key goal in myelin structural biology, they are currently being used to facilitate experimental structure solutions of full‐length CNPase and its molecular complexes at high resolution. In addition, in line with recent technical developments for Nb‐based tools (Bloch et al., 2021; Jones et al., 2023; Wu & Rapoport, 2021), we will pursue the possibility of using CNPase‐Nb complexes as fiducials for cryo‐EM experiments.

### 4.3 Structure of the CNPase‐nanobody complexes

The five NbCNPs all had different epitopes, as suggested by their varying CDR sequences. Hence, CNPase carries several potential immunogenic surfaces, which include both pockets and rather flat surfaces. Interestingly, all these epitopes were in the C‐terminal phosphodiesterase domain. It is possible that CNPase in the preparation was in an oligomeric state, which could hide the N‐terminal domain from presenting potential epitopes, or the N‐terminal domain simply was less immunogenic under these conditions. Some of the Nbs may also interact with the N‐terminal domain in addition to the main epitope in the C‐terminal domain. Our initial observation that full‐length CNPase forms oligomers in the presence of the Nbs, as well as the possible dimerisation of the catalytic domain alone in the presence of NbCNP 10E, is therefore of further interest, with regard to both structure and function of CNPase.

NbCNP 10E bound directly deep into the CNPase active site and acted as an inhibitor. Based on the crystal structure and earlier structural data, the CDR3 loop should prevent substrate binding into the active site. Therefore, it was surprising that 10E was a non‐competitive inhibitor instead of a competitive one. One clue to the mechanism came from SAXS data, indicating a 2:1 complex between the catalytic domain and NbCNP 10E. Further high‐resolution studies will be required to understand the full mechanism of inhibition, preferably in the context of full‐length CNPase.

Another intriguing case was NbCNP 7E. At first glance, it appeared not to interact with the active site. However, since activity assays indicated an increase in activity, the interactions of the nanobody with the α3‐β2 loop in the vicinity of the active site appear to be functional. The conformational change of Arg224, moving much closer to the active site, may be a means to increase catalytic efficiency of CNPase, for example, by changing the electrostatic potential in the active site—or by affecting the water network, which is important for substrate recognition and catalysis (Myllykoski et al., 2013; Raasakka et al., 2015; Raasakka & Kursula, 2014). This effect is of interest when considering the reasonably well‐defined catalytic mechanism of CNPase (Heaton & Eckstein, 1996; Myllykoski et al., 2013; Myllykoski, Raasakka, et al., 2012; Raasakka et al., 2015; Sakamoto et al., 2005) and its implications. For example, the mutation P225G in the neighbouring residue in the same loop caused a similar effect on CNPase catalytic properties as 7E (Raasakka et al., 2015); therefore, the α3‐β2 loop apparently plays a role in fine‐tuning the enzymatic reaction of CNPase. Similarly, Arg307 on the opposite side of the active‐site cavity was shown to be important for catalysis; its mutation to Gln caused an increase in both *k*_cat_ and *K*_M_ (Raasakka et al., 2015).

NbCNPs 5E, 8C, and 8D had no apparent effects on CNPase catalytic activity in the current study, when 2′,3′‐cNAPD^+^ was used as substrate. They might, however, affect activity towards larger substrates, such as RNA. Of note, all five CNPase‐Nb complexes were submitted for structure prediction in CASP15, in order to explore the ease of predicting nanobody‐target interactions and correct paratope/epitope conformations. As described (Alexander et al., 2023), one of them (complex with NbCNP 8C) could not be predicted correctly by any of the contestants. This is likely linked to the conformation of the CDR3 loop in the 8C structure, which contains a short helix held in place by a disulphide bridge.

### 4.4 Anti‐CNPase nanobodies as tools for imaging and as functional intrabodies

CNPase is one of the most commonly used markers for myelinating glia in the CNS and PNS (Huang et al., 2023; Kuhn et al., 2019; Michalski et al., 2018; Sprinkle, 1989). It is localised in non‐compact myelin areas, including cytoplasmic channels, paranodal loops, and Schmidt‐Lanterman incisures (Snaidero et al., 2017; Trapp et al., 1988). Hence, we see an interest and potential in further developing the anti‐CNPase nanobodies into versatile tools for both routine and high‐resolution imaging applications and functional studies.

Imaging of myelin is a standard tool to visualise the structure of the nervous system; however, myelin is a tightly packed structure and can generate challenges for immunolabelling due to penetration barriers. Hence, it is important to note that Nbs have broadly reported advantages compared to conventional antibodies in tissue penetration and protein‐crowded areas, and that their small size benefits super‐resolution fluorescence microscopy (D'Este et al., 2024; Mikhaylova et al., 2015; Ries et al., 2012; Sograte‐Idrissi et al., 2020). We have shown here that the anti‐CNPase Nbs are highly specific; no signal was visible by staining CNPase‐deficient nerves. Direct labelling of Nbs allows a simplified single‐step immunostaining, and their small size combined with their strong affinity provides high signal‐to‐noise, allowing clean staining on teased nerves and fast penetration on whole brain tissue sections. The strong signal and small linkage error make these Nbs ideal for molecular imaging using super‐resolution microscopy (e.g., STED). As such, the current set of NbCNPs can already function as a toolkit for high‐resolution imaging of myelinated tissue. One development option would be to combine these Nbs simultaneously, guided by the high‐resolution crystal structures, to increase the signal‐to‐noise even further. A pilot experiment here showed an increased signal when using a mixture of three labelled NbCNPs (Figure S4). Nbs targeting other myelin proteins could provide even more opportunities for high‐resolution imaging of myelinated tissue.

Intrabodies, or antibody fragments expressed inside living cells, are gaining increasing use for functional studies of target molecules (Ishizuka et al., 2022; Marschall et al., 2015; Queiroz Zetune Villa Real et al., 2023), and they are being developed towards therapeutic (Boldicke, 2022; Zhang et al., 2020) and diagnostic (Gerdes et al., 2020) applications. The anti‐CNPase Nbs could be used to modulate CNPase activity in living cells and to affect its interactions with natural partners, such as the actin cytoskeleton. We now have a structural basis for both inhibiting and slightly activating CNPase by Nbs, and the wide spread of epitopes around the CNPase molecule should help in finding and optimising nanobody binders that can block specific molecular interactions.

## 5 CONCLUDING REMARKS

We have described the development of a panel of anti‐CNPase nanobodies, which have a wide range of possible applications. Each NbCNP has a unique epitope on the C‐terminal catalytic domain of CNPase. For solving the structure of full‐length CNPase, they will be useful as crystallisation chaperones and/or cryo‐EM fiducials. The CNPase‐Nb complexes could also be developed further as a tool for the structural stabilisation of flexible proteins in structural biology. The Nbs may provide simpler alternative tools for imaging applications, including diagnostics, where CNPase is a marker for myelinating cells. Furthermore, high‐resolution imaging on myelinated tissue using advanced microscopy is possible. The expression of NbCNPs as intrabodies opens up ways of functional intervention of CNPase‐related processes, modulating CNPase catalytic activity, as well as targeting functional fusion partners to defined cellular compartments.

## AUTHOR CONTRIBUTIONS

**Sigurbjörn Markusson:** Methodology; investigation; conceptualization; validation; formal analysis; data curation; visualization; writing – original draft. **Arne Raasakka:** Investigation; validation; supervision; formal analysis; writing – review and editing; visualization; data curation; funding acquisition; project administration. **Marcel Schröder:** Investigation; validation; formal analysis; writing – review and editing. **Shama Sograte‐Idrissi:** Investigation; validation; formal analysis; writing – review and editing. **Amir Mohammad Rahimi:** Investigation; validation; formal analysis; writing – review and editing. **Ommolbanin Asadpour:** Investigation; validation; formal analysis; writing – review and editing. **Henrike Körner:** Investigation; methodology; validation; formal analysis; writing – review and editing; visualization; data curation. **Dmitri Lodygin:** Investigation; validation; formal analysis; writing – review and editing; resources. **Maria A. Eichel‐Vogel:** Methodology; investigation; validation; formal analysis; visualization; writing – review and editing. **Risha Chowdhury:** Data curation; formal analysis; validation; writing – review and editing. **Aleksi Sutinen:** Investigation; validation; formal analysis; writing – review and editing. **Gopinath Muruganandam:** Investigation; methodology; validation; writing – review and editing; data curation; resources. **Manasi Iyer:** Investigation; validation; formal analysis; writing – review and editing; supervision. **Madeline H. Cooper:** Supervision; formal analysis; validation; investigation; writing – review and editing. **Maya K. Weigel:** Investigation; validation; formal analysis; supervision; writing – review and editing. **Nicholas Ambiel:** Investigation; validation; formal analysis; supervision; writing – review and editing. **Hauke B. Werner:** Writing – original draft; project administration; methodology; conceptualization; supervision; resources; funding acquisition; visualization; validation; formal analysis; data curation. **J. Bradley Zuchero:** Conceptualization; writing – review and editing; methodology; supervision; validation; formal analysis; project administration; funding acquisition; resources; data curation. **Felipe Opazo:** Resources; supervision; formal analysis; validation; conceptualization; methodology; writing – original draft; funding acquisition; project administration; visualization; investigation; data curation. **Petri Kursula:** Conceptualization; supervision; data curation; formal analysis; validation; investigation; funding acquisition; visualization; project administration; writing – original draft; resources.

## CONFLICT OF INTEREST STATEMENT

F.O. is a shareholder of NanoTag Biotechnologies GmbH. All other authors declare no competing interests.

## Supporting information


Data S1.

## Data Availability

Structure factors and refined coordinates for the crystal structures are available at the Protein Data Bank, under the access codes 9ERT, 9ERU, 9ERW, 9ETL, 9ETJ. Raw diffraction datasets are available on Zenodo, under the DOI: https://doi.org/10.5281/zenodo.10868891, https://doi.org/10.5281/zenodo.10868769, https://doi.org/10.5281/zenodo.10868574, https://doi.org/10.5281/zenodo.10868255. All other data that support the findings of this study are available from the corresponding authors upon reasonable request. A preprint of this article was posted on BioRxiv on June 1st, 2024: https://www.biorxiv.org/content/10.1101/2024.05.25.595513v2.
