# Supplementary material for: Nanobodies against the myelin enzyme CNPase as tools for structural and functional studies
Source: J Neurochem. 2024 Dec 10;169(1):e16274. doi: 10.1111/jnc.16274 (PMC11629607; doi:10.1111/jnc.16274)
Supplement: Supplementary file 1 — Data S1. [file JNC-169-0-s001.pdf]

Supplementary Information

Nanobodies against the myelin enzyme CNPase as tools for structural and functional studies

Sigurbjörn Markusson[1], Arne Raasakka[1,2], Marcel Schröder[1], Shama Sograte-Idrissi[3], Amir Mohammad Rahimi[3], Ommolbanin Asadpour[3], Henrike Körner[4], Dmitri Lodygin[4], Maria A. Eichel-Vogel[5], Risha Chowdhury[1], Aleksi Sutinen[6], Gopinath Muruganandam[7,8], Manasi Iyer[2], Madeline H. Cooper[2], Maya K. Weigel[2], Nicholas Ambiel[2], Hauke B. Werner[5], J. Bradley Zuchero[2], Felipe Opazo[3,9,10*], Petri Kursula[1,6*]

[1]Department of Biomedicine, University of Bergen, Bergen, Norway

[2]Neurosurgery Department, Stanford University School of Medicine, Stanford, CA, USA

[3]Center for Biostructural Imaging of Neurodegeneration (BIN), University of Göttingen Medical Center, 37075 Göttingen, Germany

[4]Department for Neuroimmunology and Multiple Sclerosis Research, University of Göttingen Medical Center, 37075 Göttingen, Germany

[5]Department of Neurogenetics, Max Planck Institute for Multidisciplinary Sciences, D-37075 Göttingen, Germany

[6]Faculty of Biochemistry and Molecular Medicine & Biocenter Oulu, University of Oulu, Finland

[7]VIB-VUB Center for Structural Biology, Vlaams Instituut voor Biotechnologie, Brussels, Belgium.

[8]Department of Bioengineering Sciences, Structural Biology Brussels, Vrije Universiteit Brussel, Brussel, Belgium.

[9]Institute of Neuro- and Sensory Physiology, University Medical Center Göttingen, 37073 Göttingen, Germany

[10]NanoTag Biotechnologies GmbH, 37079 Göttingen, Germany

[*] Corresponding Authors: fopazo@gwdg.de; Petri.Kursula@uib.no

Fig. 1C top panel

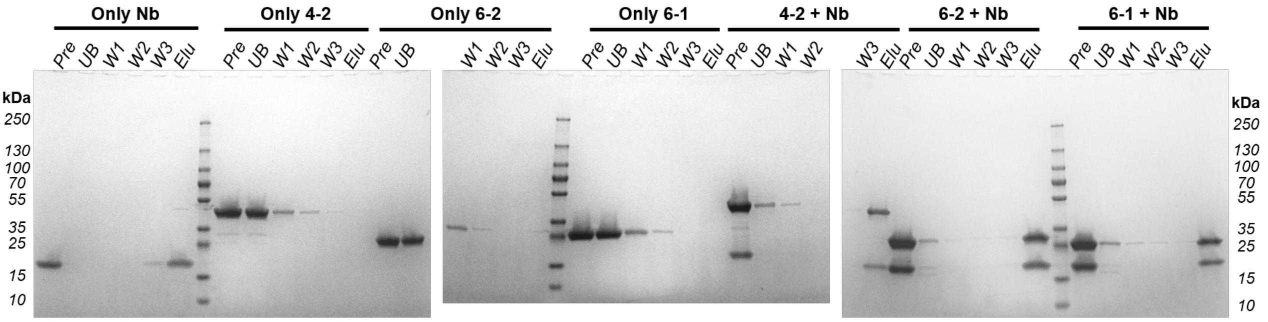

Fig. 1C bottom panel apart from last image

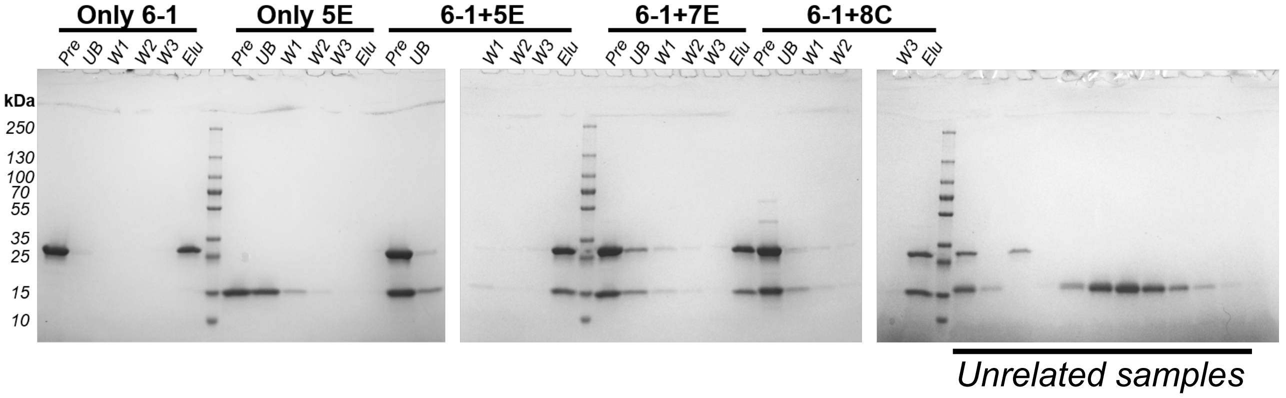

Fig. 1C bottom panel last image

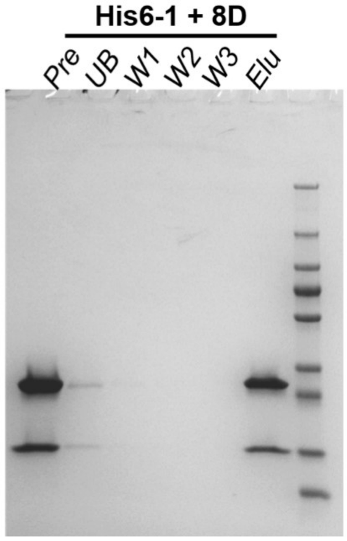

Supplementary Figure 1. Full SDS-PAGE gels for different parts of Figure 1C.

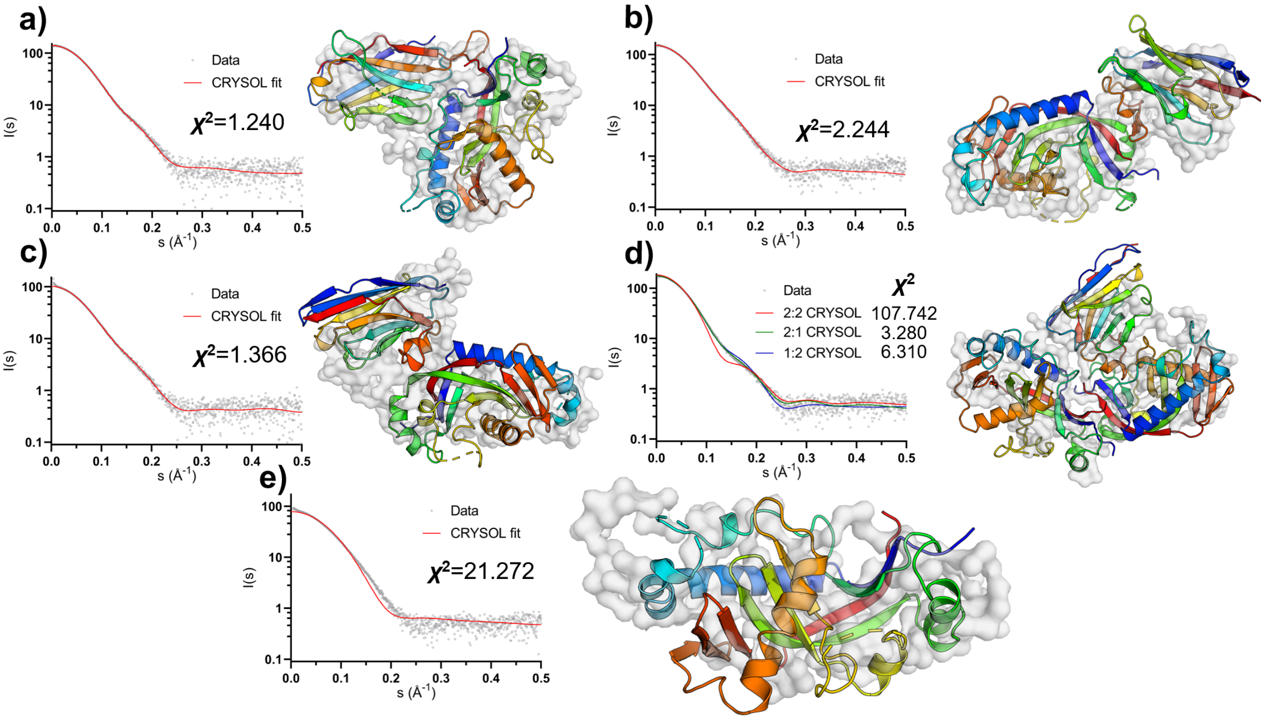

Supplementary Figure 2. SAXS data for complexes between mCNPase catalytic domain and NbCNPs 7E (a), 5E (b), 8C (c), and 10E (d). CNPase without Nbs is in panel (e). Shown to the right in each panel are the crystal structures (cartoons coloured in rainbow) superimposed onto *ab initio* models calculated using GASBOR (transparent grey surface). Each panel shows the χ_2 of the fit, where a value close to 1.0 indicates a good fit.

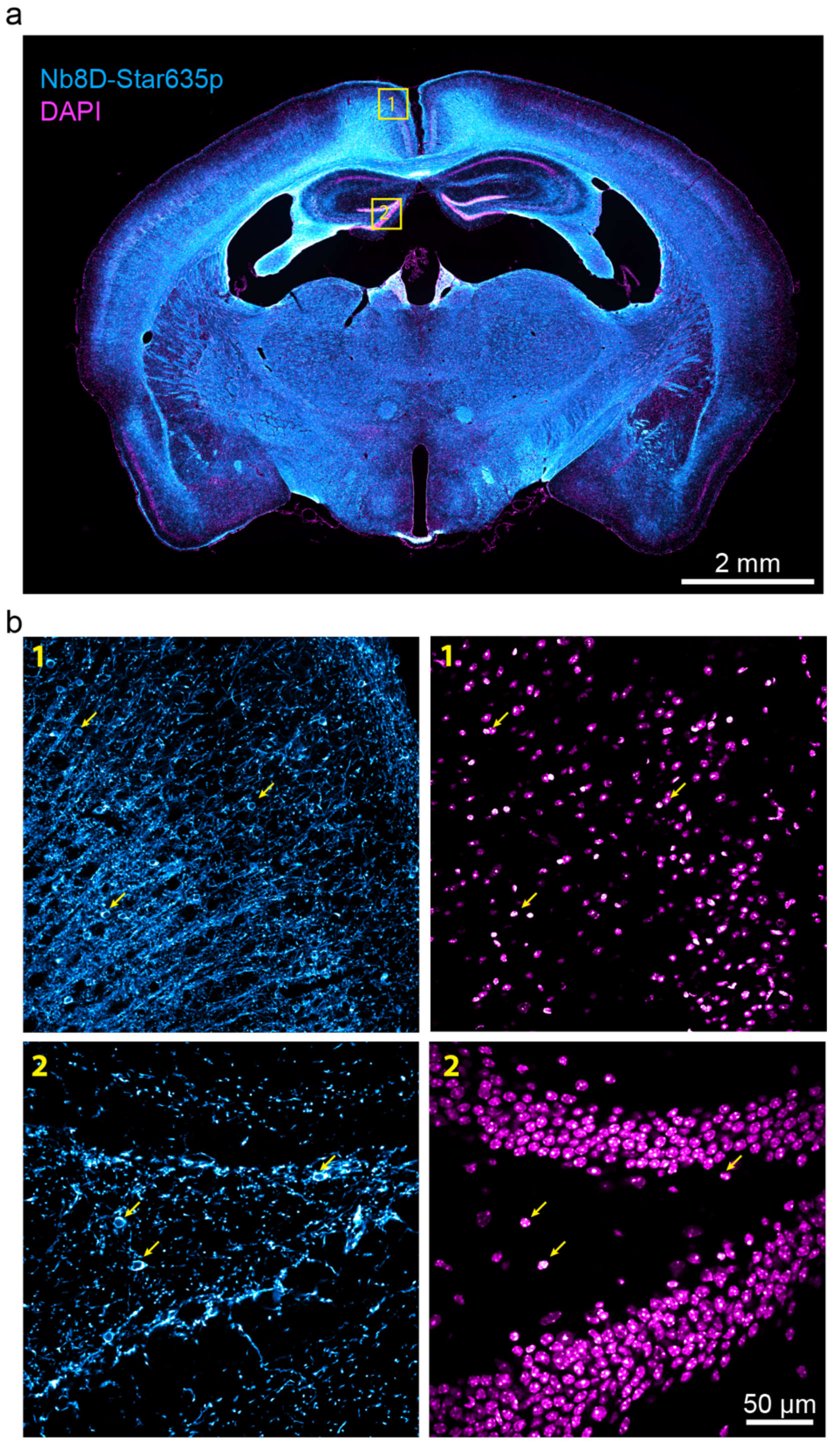

Supplementary Figure 3. High magnification confocal images on slices. (**a**) A large tiled image from Fig. 7b was further imaged under confocal microscopy in some regions. Yellow squares denote the regions that were imaged under confocal. (**b**) CNPase revealed by Nb8D-Star635p (LUT cyan-hot) reveals not only fiber-like structures but also labels the cell body of oligodendrocytes (yellow arrows) in layers on the cortex (1) as well as in the hippocampal region (2).

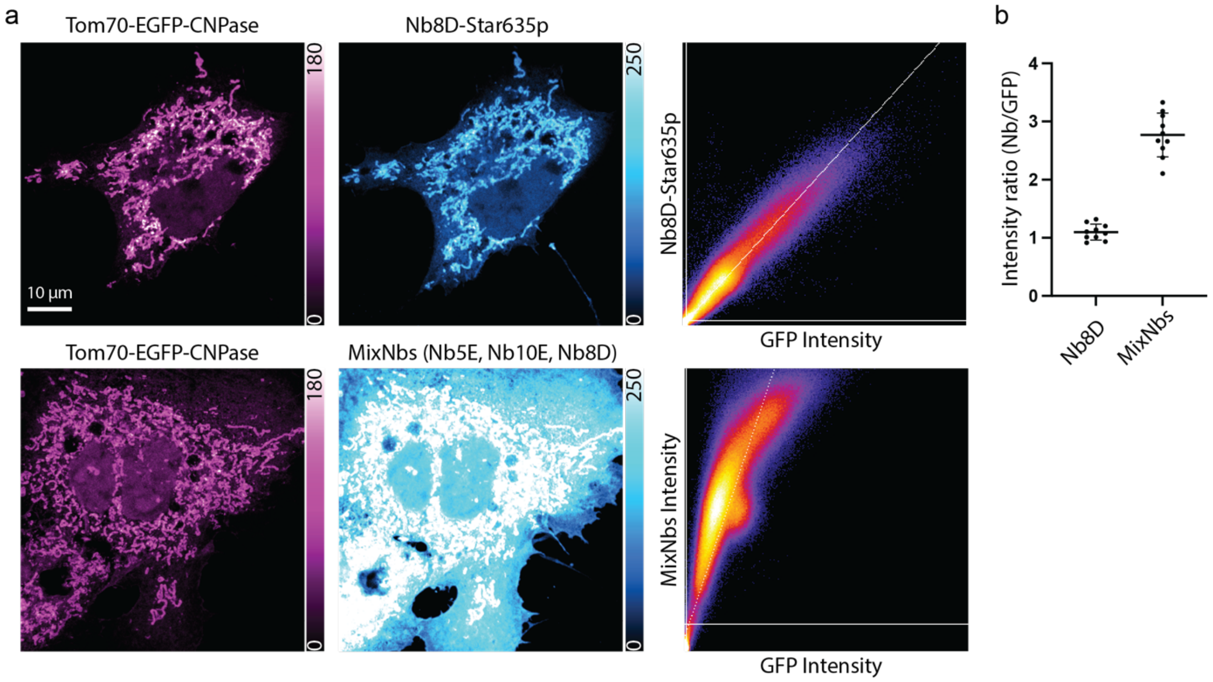

Supplementary Figure 4. COS-7 cells were transfected with Tom70-EGFP-CNPase and immunoassayed using 10 nM of NbCNP-8D conjugated with a single Star635p fluorophore or with a mixture of Nb8D, Nb5E and Nb10E all at 10 nM (MixNb), each with a single Star635p. (**a**) Exemplary confocal images of the two staining conditions: All images were acquired with the same settings and scaled equally for direct comparison. GFP signal (LUT: magenta-hot) looks comparable between the two conditions; however, the signal on the MixNb staining is clearly brighter if scaled as scaled the Nb8D-Star635 (LUT: Cyan hot) as clearly observed in the intensity correlation graph (correlation of the entire image per pixel). (**b**) Intensity correlation normalized by the intensity of EGFP. Every EGFP is fused to a single CNPase, thus if recognized by a single Nb, it gives a particular ratio; however, if the CNPase is now decorated by 3x Nbs and each carries a single fluorophore, it is expected that the ratio is three times the one obtained by a single Nb. The graph displays the average of 10 cells (N =10) and the standard deviation.

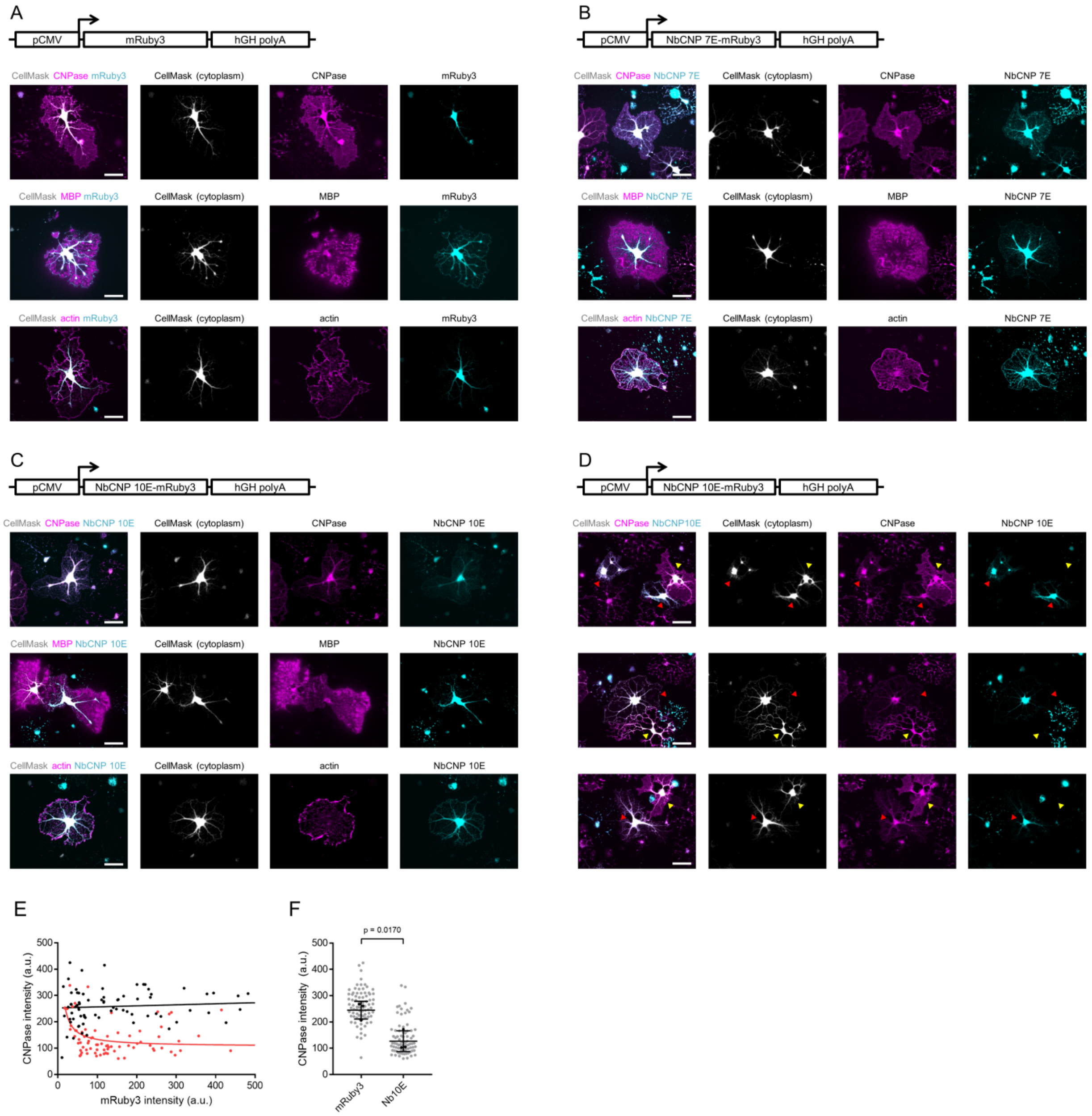

Supplementary Figure 5. Expression of NbCNPs as intrabodies fused to mRuby3 in cultured oligodendrocytes. A. mRuby3 alone control. B. NbCNP 7E. C. NbCNP 10E. D. NbCNP10E shows reduced staining for CNPase with conventional antibodies. All scale bars are 50 µm. E. mRuby3 vs. CNPase staining intensities for mRuby3 alone control (black dots) and NbCNP 10E (red dots). Lines denote non-linear regression fits to data points (line colors are matched with dot colors). F. CNPase staining intensities. Means of individual biological replicates (n = 3) are plotted as black dots and values from individual cells as grey dots. Statistical significance was calculated with Student's t-test (two-tailed; p = 0.0170, t = 3.94, df = 4) using the means of biological replicates (n = 3).

Supplementary Table 1. Mass spectrometry analysis of the NbCNPs. M_{mi} denotes monoisotopic mass and (S-S) a disulfide. Measurement of MaBP-8D was attempted, but the protein degraded during transport to the MS facility. The NbCNP 8D identity was confirmed by the crystal structure.

Protein	Calculated M_{mi} (Da)	Measured M_{mi} (Da)	Peak abundance (%)	Mass change (Da)	PTM
Nb 7E	13936.59	13936.61	66.25	+0.02	None
		13935.62	29.00	-0.98	
Nb 5E	14243.53	14241.56	52.18	-1.97	1x(S-S)
		14239.53	26.27	-4.00	2x(S-S)
Nb 8C	14079.53	14075.51	94.12	-4.02	2x(S-S)
Nb 10E	14428.81	14428.83	99.81	+0.02	None

Supplementary Table 2. Cultured oligodendrocyte cell amounts for each biological replicate.

	mRuby3			Nb7E			Nb8D			Nb10E		
n	anti-CNP	anti-MBP	phalloidin	anti-CNP	anti-MBP	phalloidin	anti-CNP	anti-MBP	phalloidin	anti-CNP	anti-MBP	phalloidin
1	44	10	10	20	10	10	30	10	10	30	15	10
2	30	10	10	30	10	10	30	10	10	39	15	10
3	6	20	10	30	10	10	20	10	10	11	15	10
Total cells	80	40	30	80	30	30	80	30	30	80	45	30

Supplementary Table 3. ANOVA statistics report related to Fig 9B.

Data sets analyzed	A : mRuby:	B : Nb7E	C : Nb8D	D : Nb10E				
ANOVA summary								
F	15.5							
P value	0.0011							
P value summary	**							
Significant diff. among means (P < 0.05)?	Yes							
R square	0.8532							
Brown-Forsythe test								
F (DFn, DFd)	0.6021 (3, 8)							
P value	0.6317							
P value summary	ns							
Are SDs significantly different (P < 0.05)?	No							
Bartlett's test								
Bartlett's statistic (corrected)								
P value								
P value summary								
Are SDs significantly different (P < 0.05)?								
ANOVA table	SS	DF	MS	F (DFn, DF	P value			
Treatment (between columns)	0.07217	3	0.02406	F (3, 8) = 1	P=0.0011			
Residual (within columns)	0.01242	8	0.001552					
Total	0.08459	11						
Data summary								
Number of treatments (columns)	4							
Number of values (total)	12							
Number of families	1							
Number of comparisons per family	3							
Alpha	0.05							
Dunnett's multiple comparisons test	Mean Diff.	95.00% CI	Significant'	Summary	Adjusted P	?-A		
Nb7E vs. mRuby3	0.1215	0.02889 to	Yes	*	0.0137	B	Nb7E	
Nb8D vs. mRuby3	0.1884	0.0958 to (	Yes	***	0.001	C	Nb8D	
Nb10E vs. mRuby3	0.1913	0.09869 to	Yes	***	0.0009	D	Nb10E	
Test details	Mean 1	Mean 2	Mean Diff.	SE of diff.	n1	n2	q	DF
Nb7E vs. mRuby3	0.7637	0.6422	0.1215	0.03217	3	3	3.778	8
Nb8D vs. mRuby3	0.8306	0.6422	0.1884	0.03217	3	3	5.858	8
Nb10E vs. mRuby3	0.8335	0.6422	0.1913	0.03217	3	3	5.948	8
